# SIREs 3.0, an improved RNA prediction tool for iron-responsive elements

**DOI:** 10.1093/nar/gkaf390

**Published:** 2025-05-05

**Authors:** Clara Suárez-Quintana, Mar Navarro-Padilla, Uciel Chorostecki, Mayka Sanchez

**Affiliations:** Department of Biomedicine, Iron metabolism: Regulation and Diseases Group, Universitat Internacional de Catalunya (UIC), Sant Cugat del Vallès 08195, Spain; Department of Biomedicine, Computational RNA Biology Group, Universitat Internacional de Catalunya (UIC), Sant Cugat del Vallès 08195, Spain; BloodGenetics S.L. Diagnostics in Inherited Blood Diseases, Esplugues de Llobregat 08950, Spain; Department of Biomedicine, Computational RNA Biology Group, Universitat Internacional de Catalunya (UIC), Sant Cugat del Vallès 08195, Spain; Department of Biomedicine, Iron metabolism: Regulation and Diseases Group, Universitat Internacional de Catalunya (UIC), Sant Cugat del Vallès 08195, Spain; BloodGenetics S.L. Diagnostics in Inherited Blood Diseases, Esplugues de Llobregat 08950, Spain

## Abstract

Iron-responsive elements (IREs) are *cis*-acting regulatory RNA motifs that bind to iron regulatory proteins (IRP1 and IRP2), playing an essential role in the post-transcriptional regulation of genes involved in iron metabolism. Disruptions in this IRP/IRE regulatory system have been linked to several human diseases. SIREs (searching for IREs) is a web-server tool designed to predict IREs in nucleotide sequences. Here, we present SIREs 3.0 webserver, an improved new version built on a Flask-based framework, replacing the previous Perl backend to improve interconnectivity with other services. This upgrade introduces three novel input methods—batch, transcript, and gene modes—to cover researchers’ needs, from large-scale analysis to single-gene queries. By integrating with NCBI and Ensembl APIs, SIREs 3.0 fetches genomic data to improve predictions with novel features, such as the location of the IREs in the transcript. Other novelties include novel IRE motifs based on *in vivo* verified data, broadening the scope of IRE detection. The scoring system has been refined with empirical data and enhanced graphical representations of predicted IREs have been added to the output. The interface is now more responsive and accessible across all devices. SIREs 3.0 can be accessed at: https://www.sires-webserver.eu.

## Graphical Abstract

**Graphical Abstract:**
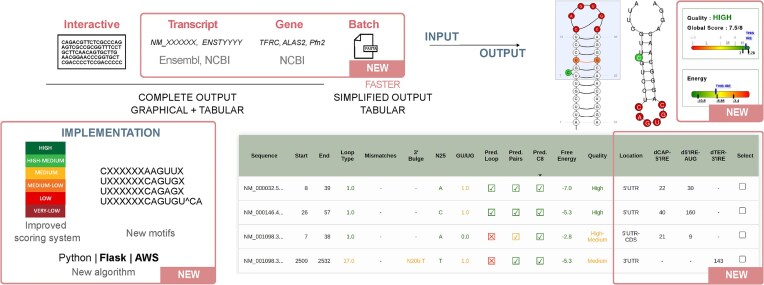

## Introduction

Iron is an essential biomineral subject to complex regulatory mechanisms to maintain its homeostasis. Cellular iron metabolism is post-transcriptionally regulated by an RNA-protein binding mechanism: the iron regulatory protein/iron-responsive element (IRP/IRE) system. IREs are RNA sequence-structure motifs, typically found in the untranslated regions (UTRs) of iron metabolism messenger RNAs (mRNAs). IREs are characterized by a 6-nucleotide apical loop (5′-CAGWGH-3′; W: A, U/H: A, C, U) on a stem of five paired nucleotides, a small asymmetrical bulge with an unpaired cytosine on the 5′ strand of the stem and an additional lower stem of variable length [1–3]. IRPs are bifunctional proteins that, under iron-starvation conditions, bind IREs to control the expression of their target mRNAs [3].

Since the identification of the IRE in ferritin mRNAs [4, 5], additional IREs have been validated, primarily in genes regulating iron metabolism. These include genes involved in iron acquisition, such as *TFRC* (transferrin receptor) [6] and *SLC11A2* (divalent metal transporter 1) [7], iron export, including *SLC40A1* (ferroportin) [8], and iron/oxygen sensing, such as *EPAS1* (hypoxia-inducible factor 2α) [9]. Additionally, in 2017, a noncanonical IRE was identified in the mRNA of profilin2 (*Pfn2*), an actin-binding protein [10], demonstrating that functional IRE-like structures can extend beyond the classical apical loop configuration. This discovery expanded the concept of IRE diversity, indicating that alternative RNA structures may also regulate gene expression in response to iron.

From the outset, identifying and validating novel IREs has relied on a combined approach of computational prediction and experimental validation [11]. To support the initial discovery phase, our group developed the searching for iron-responsive elements (SIREs) web server in 2010 [12], a versatile tool for predicting potential IREs in RNA and DNA sequences and ranking them according to a confidence score. Over the years, SIREs software has become a valuable resource for the iron research community, serving as a key starting point for characterizing new IREs, including those in *BDH2* [13] and *CD63* [14].

Since its first release, SIREs has assisted researchers by employing a sequential algorithm that first identifies an apical loop matching one of 18 defined motifs. It then refines predictions by applying penalties for mismatches and bulges in the stem, while also assessing concordance with RNA secondary structure predicted by RNAfold [15].

Despite significant advancements in research on the IRP/IRE system, SIREs has remained the community’s preferred tool for IRE prediction. To enhance its functionality and usability while meeting evolving research needs, we have developed SIREs 3.0, featuring a complete overhaul of both the back-end and front-end. The source code has been migrated to Python, and the application is now deployed using the Flask framework on Amazon Web Services (AWS). This latest version introduces key improvements, including integration with NCBI and Ensembl, an expanded motif database, three new input modalities, a redesigned interface, and enhanced graphical outputs for a more intuitive user experience.

## Server implementation

The source code of SIREs 3.0 was migrated from Perl to Python3 to ensure easier maintenance and seamless integration with newly developed modules. The Flask web framework was chosen for its simplicity, scalability, and well-structured architecture, providing a robust foundation for the application. To optimize performance and reliability, the application was deployed on AWS, specifically on an EC2 instance with 2vCPUs and 8 GiB of memory. Additionally, a backup system was implemented using an S3 instance. The deployment leverages NGINX as a reverse proxy server and Gunicorn as the WSGI application server, enhancing efficiency and load management.

SIREs 3.0 web server is compatible with all major web browsers, including Opera, Edge, Google Chrome, Safari, and Firefox across the major platforms (Windows, MacOS, and Linux). Submitted jobs are securely stored for seven days before automatic deletion.

## Novel features present in SIREs 3.0

### Integration of NCBI and Ensembl REST APIs

A key enhancement in SIREs 3.0 is the integration with the NCBI [16] and Ensembl [17] REST APIs. This integration allows the program to make internal calls to these databases, retrieving valuable transcript, and gene data. As a result, users benefit from improved functionality, including novel input methods and more comprehensive output generation, which significantly enhances the overall user experience.

### Additional input methods

The original SIREs version of SIREs accepted input data in the form of FASTA sequences, with a maximum length of 500 000 residues, which had to be pasted into a text box for submission [12]. In this updated version, this original mode is now referred to as “interactive mode”, which still supports the submission of up to 50 000 residues at a time. Additionally, three new input modes have been implemented, offering greater flexibility and functionality (Table 1). Importantly, due to the way IREs are detected—requiring not only the core element (∼30 nt) but also flanking regions for accurate structure prediction and output formatting—very short sequences may result in missed predictions. SIREs accepts inputs as short as 19 nucleotides [or 20 nucleotides when a 3′ bulge is present, as in the case of the HIF2*α* (*EPAS1*) IRE], although structure prediction may be less reliable at this lower limit. For optimal results, we recommend input sequences of at least 31–32 nucleotides to provide sufficient structural context.

**Table 1.** SIREs v1.0, 2.0 and 3.0 input and output modes

Mode	Version	Input	Output	Average computational time (s)^a^
Interactive	v1.0–2.0	FASTA sequence < 50 000 nts	Graphical + tabular	30
Transcript	v3.0	NCBI and Ensembl transcript IDs	Graphical + tabular	2
Gene name	v3.0	Official gene name	Graphical + tabular	65
Batch	v3.0	File	Tabular	15

^a^For interactive mode, the average computation time is based on 10 runs with input sequences of approximately 50 000 nucleotides (0.5 Mb). For transcript and gene modes, the average computation time is based on 10 separate submissions for each. For batch mode, the average computation time is calculated for 10 runs with input sequences of 50 000 nucleotides (0.5 Mb).

#### Batch mode

This mode allows the processing of thousands of sequences more efficiently by generating a simplified output without graphical elements. Users can then select up to 10 sequences from the batch results and re-submit them through the interactive server for a detailed output.

#### Transcript mode

In this mode, users can input an official transcript ID from NCBI (NM_, XM_, and NR_) or Ensembl stable ID prefixes (https://www.ensembl.org/info/genome/stable_ids/prefixes.html). SIREs then retrieves the corresponding sequence and genomic annotations internally to run the predictions.

#### Gene mode

In this mode, users can enter an official gene name and select the organism of interest from a dropdown menu. SIREs will then internally retrieve the relevant NM_, XM_ and NR_ transcripts from NCBI for that gene. To ensure optimal performance and maintain this mode as a fast prediction option, only NCBI-curated transcripts are included, preventing server overload.

Each input mode in SIREs 3.0 comes preloaded with at least one example, which can be easily loaded and submitted with a single click for demonstration purposes.

### Expanded set of motifs

**Figure 1.**
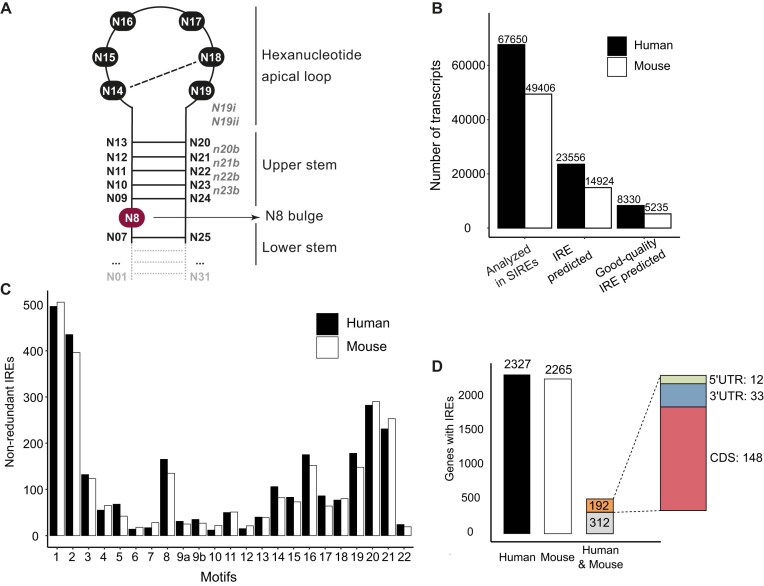
SIREs web server v3.0. (**A**) Schematic representation of the IRE structure. The region used by SIREs for prediction—comprising the loop, upper stem, and the N07–N25 pair from the lower stem—is shown with a solid line. The remaining portion of the lower stem, indicated by a dotted line, is not directly used for IRE prediction but contributes to the folding prediction of the full secondary structure (and the free energy of the full IRE structure), therefore influencing the IRE quality score. Although the lower stem exhibits greater variability across functional IREs, it is included due to its important role in IRE function. (**B**) Bar graph showing the analysis of NCBI (NM_) human and mouse transcripts using SIREs software. The first two columns represent the total number of NCBI transcripts processed. The third and fourth columns indicate the number of transcripts in which at least one IRE was predicted. The fifth and sixth columns represent the number of transcripts containing at least one good-quality IRE (i.e. classified into quality categories: high, high-medium, and medium). (**C**) Motif distribution of good-quality IREs in nonredundant transcripts. Redundancy was removed by retaining only one occurrence of identical IREs predicted across multiple transcripts of the same gene; distinct IREs within the same gene were kept. (**D**) Comparison of nonredundant transcripts containing an IRE in human and mouse. The first two bars represent the number of nonredundant transcripts present in human (left) and mouse (middle). The third bar indicates the number of transcripts that contain an IRE in both species. The upper fraction within this third bar highlights the subset of transcripts that share the same IRE motif and localization in both species, suggesting a conserved IRE.

SIREs’ algorithm screens input sequences to identify RNA motifs, starting with 18 IRE motifs from our previous work [12] (motifs 1–18, Fig. 1A and Table 2), which were originally derived from canonical IREs and motifs identified in systematic evolution of ligands by exponential enrichment (SELEX) experiments [18, 19]. Based on experimental and literature data, we have expanded the motif set by four additional IREs (Table 2, motifs 19–22) and expanded motif 9 into 9a and 9b (Table 2, motifs 9a and 9b). Motif 19 is based on our previous experimental work, where we identified a novel IRE in the 3′UTR of the *Pfn2* mRNA [10]. This new IRE deviates from canonical IREs, featuring an atypical apical loop with changes at positions N14 (A instead of C) and N18 (U instead of G), while retaining a cytosine at N8 (Fig. 1A and Table 2). Motifs 20–22 are based on the findings from a 2001 study by Meehan and Connel, which explored IRE structural preferences. These motifs feature a U8 bulge instead of the canonical C8 bulge. Notably, motif 22 also includes two additional nucleotides (CA) extending from the apical loop, designated as N19i and N19ii (Fig. 1A and Table 2). Motif 9 was already present in SIREs v1.0 and v2.0. However, we identified it as a complementary variant of motif 19, featuring a U at position N14 and an A at position N18. SIREs v1.0 and v2.0 allowed only U/C at position N19, whereas motif 19 permits any nucleotide at this position. To account for this flexibility, we expanded motif 9 into two variants: 9a (corresponding to motif 9 in v1.0 and v2.0) and 9b (allowing G /A at N19) (Table 2).

**Table 2.** Motifs used in SIREs software

Version	Motif	N8	N14–N19	Position N14–N18	N19i–N19ii
v1.0–2.0	1 (classical)	C	CAGUG(A/C/G/U)	C-G	–
v1.0–2.0	2 (classical)	C	CAGAG(A/C/U)	C-G	–
v1.0–2.0	3	C	CUGUG(C/U)	C-G	–
v1.0–2.0	4	C	CCGUG(A/C/U)	C-G	–
v1.0–2.0	5	C	CCGAGA	C-G	–
v1.0–2.0	6	C	CUUAGC	C-G	–
v1.0–2.0	7	C	CAAUGC	C-G	–
v1.0–2.0	8	C	CAGGG(A/C/G/U)	C-G	–
v1.0–2.0	9a	C	UAGUA(C/U)	U-A	–
v3.0	9b	C	UAGUA(A/G)	U-A	–
v1.0–2.0	10	C	UAGGAU	U-A	–
v1.0–2.0	11	C	UAGAA(C/U)	U-A	–
v1.0–2.0	12	C	UAGCAG	U-A	–
v1.0–2.0	13	C	GAGUC(A/G)	G-C	–
v1.0–2.0	14	C	GAGCC(A/G)	G-C	–
v1.0–2.0	15	C	GAGAG(G/U)	G-G	–
v1.0–2.0	16	C	GGGAG(A/C/G/U)	G-G	–
v1.0–2.0	17	C	GAGUG(A/U)	G-G	–
v1.0–2.0	18	G	CAGUGA	C-G	–
v3.0	19	C	AAGUU(A/C/G/U)	A-U	–
v3.0	20	U	CAGUG(A/C/G/U)	C-G	–
v3.0	21	U	CAGAG(A/C/G/U)	C-G	–
v3.0	22	U	CAGUG(A/C/G/U)	C-G	CA

### Refinement of the scoring system

The scoring system has been refined after reviewing existing literature, including IRE mutagenesis experiments [20, 21] and patient mutations affecting IREs with phenotypic implications [22–24]. Notably, we observed that the same N07–N25 IRE position is altered in two diseases (as a modifier for erythro-protoporphyria and in hereditary hyperferritinemia with cataracts syndrome) across two different IREs (*ALAS2* and *FTL*, respectively) [24–26]. Based on this observation, we increased the penalty for changes at the N07–N25 IRE position and validated the adjustment. This modification led to a shift in the scoring for the *ALAS2* mutant IRE and *FTL* mutant IRE from high to low and medium-low (Supplementary Fig. S1A).

Additionally, the scoring scale has been expanded from a three-category system (High, Medium, and Low) to a six-category system (High, High-medium, Medium, Medium-low, Low, and Very low). This enhancement allows users to differentiate the quality of predicted IREs (Supplementary Fig. S1B) more precisely.

### Extended output

#### IRE location within transcript

The regulatory effect of IRP binding on mRNA depends on the IRE location in the transcript. When IRP binds to a 5′UTR IRE, it impedes mRNA translation, whereas binding to a 3′UTR IRE stabilizes the mRNA. Therefore, the position of the predicted IRE within the transcript is crucial, as it determines its functional outcome.

IRE location is retrieved by SIREs 3.0 in Transcript and Gene modes. In Interactive and Batch modes, the sequence identifier must start with the official transcript ID; otherwise, genomic annotation will not be retrieved, and the location cannot be computed.

SIREs 3.0 considers five locations: 5′UTR, 5′UTR-CDS, CDS, CDS-3′UTR. We introduced the 5′UTR-CDS and CDS-3′UTR categories to accommodate IREs, like *ACO2*, that span multiple transcript regions (i.e. IRE *ACO2* is partly in the 5′UTR and partially in the coding region).

To compute this, we use the middle position of the IRE, either position N16 or N17, depending on the type of IRE (with or without a 3′ bulged nucleotide). For an IRE near the 5′UTR, if the distance from this nucleotide to the first nucleotide of the starting codon (A + 1 in most transcripts) is < |15|, it is classified as a 5′UTR-CDS IRE. Similarly, for an IRE near the 3′UTR, if the distance from the IRE middle nucleotide to the last nucleotide of the stop codon is < |15|, it is classified as a CDS-3′UTR IRE.

#### Distance to key transcript elements

In line with prior functionality, we now compute three distances for IREs located within the UTRs, as proximity to key transcript structures, such as the CAP structure, has been suggested to influence IRE-IRP binding activity [27]. For an IRE in the 5′UTR or 5′UTR-CDS boundary, we calculate two distances: from the IRE midpoint to the translational start site (d5′IRE-AUG) and to the m7G-cap (dCAP-5′IRE). For an IRE in the 3′UTR or CDS-3′UTR, we compute the distance from the terminal codon to the IRE midpoint (dTER-3′IRE). Knowing these distances allows us to determine the IRE’s position relative to the translation start site, m7G-cap structure, and terminal stop codon.

#### Implementation of graphical representations of quality and free energy predictions

To assist in interpreting prediction quality, the results page now features two sections framed within rectangles (Supplementary Fig. S2). The top frame displays the quality category (High, High-medium, Medium, Medium-low, Low, Very low), the score, and a color-coded bar ranging from red to green on a scale of negative values to 8. We computed the average SIRE scores for 28 validated and gold-standard IREs (motifs 1, 2, and 19): 14 from human genes, 13 from mouse genes, and 1 from drosophila genes (Supplementary Table S1). The average predicted score for these IREs is 7.36/8, with *CDC14A*, *EPAS1*/*Epas1*, *Fth1*, *Ftl1*, *SLC40A1*/*Slc40a1*, *TFRC*/*Tfrc* (A, B, E, and D) and drosophila *SdhB* IREs scoring 8, and the lowest score being 5.50/8 for *SLC11A2*/*Slc11a2*. The lower frame shows free deltaG energy, represented by a color-coded bar from green to red, with the worst possible free deltaG energy of 0. The average free energy of the 28 gold-standard IREs is −7.11 kcal/mol, with *ACO2*/*Aco2* IREs having the highest free energy (−2.80 kcal/mol) and *TFRC*/*Tfrc* IRE E having the lowest (−11.70 kcal/mol). The IRE under study is marked as a blue line on both colour bars.

### Interface redesign and responsiveness

SIREs interface has been completely renovated. The main page has been updated to improve navigation and facilitate the transition between input modes, incorporating help buttons to assist users with the new features (Supplementary Fig. S3). Concordantly, we also renewed the results page, to maintain the aesthetics and facilitate the understanding of the output.

## Comparison to other services and performance

As for the existence of alternatives to SIREs, we are only aware of two other bioinformatic tools, RNAAnalyzer and RNAMotif, which were released >15 years ago and have not undergone significant updates over the years [28, 29]. To reassess our program’s performance, we conducted a new benchmarking analysis, similar to the initial evaluation done in Campillos *et al.*, 2010. We used a dataset of 56 positive controls (20 gold-standard IREs and 36 mRNAs binding IRP1/IRP2; [21]) and 150 curated mouse transcripts as negative controls, randomly selected from NCBI, excluding duplicates and known IRE-containing sequences (Supplementary Table S2). For each software, we evaluated sensitivity, specificity, balanced accuracy and precision (Supplementary data – Benchmarking analysis). Balanced accuracy was included to account for the class imbalance between positive and negative controls in the benchmarking dataset.

All tools were evaluated under identical conditions for a fair comparison. To achieve this, we computed overall performance metrics for SIREs by pooling predictions across confidence categories, as the other tools do not differentiate between these categories.

SIREs demonstrated superior sensitivity (75.00%) compared to RNAMotif (32.08%) and RNAAnalyzer (33.96%), detecting more true positives overall (Table 3). This confirms SIREs’ strength in recovering a broad range of biologically plausible IREs—including noncanonical motifs missed by other methods. However, this comes at the cost of reduced precision (50.60%) and specificity (72.77%), likely due to its broader motif recognition. By identifying up to 22 distinct motifs, SIREs may introduce some noise but enables a more comprehensive exploration of potential biological patterns.

**Table 3.** Performance assessment of SIREs and two additional IREs predictive softwares

			SIREs 3.0			
				Quality categories		
	RNAMotif	RNAAnalyzer	Overall	*High*	*Medium*	*Low*
Sensitivity	32.08	33.96	75.00	52.83	22.64	26.41
Specificity	97.33	98.00	72.77	98.67	89.33	77.33
Precision	80.95	85.71	50.60	93.30	42.86	29.17
Balanced accuracy	65.7	65.98	73.83	75.74	55.98	51.87

Additionally, we also provide, for SIREs, metrics by confidence levels. To ensure reliable evaluation, we grouped SIREs predictions into three pooled confidence categories: high confidence, combines “high” and “high-medium” predictions; medium confidence, combines “medium” and “medium-low” predictions; and low confidence, which combines “low” and “very low” predictions. This pooling strategy improves interpretability and yields more robust estimates, especially given that some categories are sparsely populated.

High-confidence predictions achieved excellent precision (93.30%) and specificity (98.67%) with moderate sensitivity (52.83%), providing a reliable set of candidates for experimental validation. As expected, medium- and low-confidence groups demonstrated lower precision and sensitivity, reflecting the uncertain biological support for these motifs. These lower-confidence predictions should be interpreted with care but may include novel candidates worth exploring. The unpooled metrics—based on the original six confidence categories—are also provided in Supplementary Table S3 and reflect a gradual performance drop in medium- and lower-confidence bins, underperforming if compared against RNAMotif and RNAAnalyzer, but consistent with SIREs’ broader detection scope.

While older tools remain more conservative, SIREs 3.0 is explicitly designed for exploratory analysis, enabling the discovery of potential novel IREs—including those with atypical structures like 3′ bulges or noncanonical loops. This broader scope may introduce more false positives but empowers researchers to investigate a wider landscape of regulatory elements.

## Case study

### Screening of human and mouse protein-coding transcripts using batch mode

To highlight the new functionalities of SIREs, we conducted a large-scale screening of all experimentally validated human and mouse protein-coding transcripts (NM_) from NCBI. We downloaded the RefSeq data and processed it as indicated in Supplementary data – Case study. Using SIREs, we analyzed 67 650 human and 49 406 mouse sequences. Our tool identified 23 556 human transcripts and 14 924 mouse transcripts harbouring at least one predicted IRE. When focusing on good-quality IREs (classified as High, High-medium, or Medium quality sub-category), 12.13% of human transcripts (8330) and 10.60% of mouse transcripts (5235) were predicted to contain an IRE (Fig. 1B). This initial analysis did not account for redundancies, meaning the total transcript count may include the same IRE appearing in different isoforms of the same gene. For subsequent analyses (Fig. 1C and D), we focused on good-quality IREs (quality sub-categories High, High-medium, and Medium), with redundancy across transcripts removed at the gene level to ensure that each IRE was counted only once. When identical IREs were predicted in multiple transcripts of the same gene, a single occurrence was retained, while distinct IREs within the same gene were kept (Supplementary data – Obtention of nonredundant IREs).

Among the predictions, SIREs 3.0 successfully identifies all well-characterized, validated IREs, including those found in *FTH1*, *FTL*, *TFRC*, *ALAS2*, *ACO2*, *SLC11A2*, *SLC40A1*, and *EPAS1* (and their mouse orthologs). Additionally, validated IREs in *PFN2/Pfn2 and CDC14A* (human) are also detected. Moreover, high-scoring IREs were found in 25 mouse genes previously shown to bind IRP1 or IRP2 *in vitro* by RNA immunoprecipitation with recombinant proteins, as reported previously [21], providing further support for the biological relevance of SIREs predictions.

Most of the good-quality IREs were located within coding regions, followed by the 3′ UTR and the 5′ UTR. A smaller fraction appeared in the 5′UTR-CDS and in the CDS-3′UTR (Supplementary Fig. S4A). However, when normalized by length, 5′ UTRs emerged as the most enriched region for IRE predictions (Supplementary Fig. S4B). Regarding motif distribution, canonical motifs 1 and 2 accounted for 33.16% of human and 32.10% of mouse IRE predictions. Other notable motifs included motifs 20 and 21 (for both human and mouse) and motifs 8, 16, and 19 (Fig. 1C).

We also examined the conservation of IREs between human and mouse, as their preservation may suggest a shared regulatory role. Among the good-quality IREs, SIREs identified 504 nonredundant transcripts that contained an IRE both in the human transcript and its orthologous mouse counterparts (Fig. 1D). Of these 504 transcripts, 192 transcripts contained the same IRE motif and in the same transcript region, indicating the presence of a conserved IRE in both species (Fig. 1D).

The majority of these conserved IREs were found in the CDS (148/192), but a smaller subset was identified in the 5′ UTR [12] and the 3′ UTR [30] (Fig. 1D and Supplementary Table S4). Overall, we demonstrate that SIREs software provides a valuable starting point for more in-depth analyses of predicted IRE. Our tool serves as a useful resource for guiding experimental research and facilitates the identification of potential targets for further functional studies.

### Superior detection of reported IREs by SIREs

To further validate the performance of SIREs v3.0 we conducted a case study using a curated set of 26 IREs reported in the literature [6–11, 13, 14, 30–43]. SIREs v3.0 successfully detected 80.77% (21/26) of these IREs, outperforming RNAMotif (46.15%) and RNAAnalyzer (38.46%) (Supplementary Table S5). Importantly, SIREs v3.0 was uniquely capable of systematically identifying atypical IREs with a 3′ bulge, such as the ones in *SLC11A2* (DMT1) and *EPAS1* (HIF-2α) mRNAs [7, 9], as well as a functional IRE in the *PFN2* 3′UTR previously confirmed to regulate iron metabolism *in vivo* [10]. These results demonstrate the enhanced sensitivity and broader detection capabilities of SIREs v3.0 compared to existing prediction tools.

## Summary

Significant progress has been made in the fields of iron metabolism and RNA prediction, and our SIREs software continues to be the leading tool for predicting IREs and serving as a foundation for experimental validation. We present SIREs 3.0, which introduces key updates to advance iron metabolism research. With an upgrade to Python3 and hosting on AWS, the software is now faster, more reliable, and easier to maintain. Integration of NCBI and Ensembl APIs has streamlined data retrieval and provided flexible input options for diverse research needs. The expanded motif set, incorporating newly validated IREs and enhanced scoring, improves prediction accuracy—particularly for noncanonical IRE structures that were previously difficult to detect. For example, SIREs 3.0 successfully identifies IREs with 3′ bulges (such as in *EPAS1*) [9] and noncanonical loops (as in the validated *Pfn2* IRE) [10], highlighting a key strength of the tool in uncovering potentially functional elements that would otherwise be missed. Additional features, such as transcript localization and distance metrics to key elements, offer more detailed insights into the functional roles of IREs. SIREs 3.0 also boasts a user-friendly interface and enhanced visual outputs, making it more accessible and informative for users.

SIREs detects all well-known IREs, including those in the ferritins, transferrin receptor, *ALAS2*, *ACO2*, and others, and its predictions can guide targeted experimental studies. However, it is important to note that only a small number of IREs have been experimentally validated to date, and some predicted elements may represent false positives. We believe that maintaining a broader predictive scope is essential, as stringent filtering could exclude biologically meaningful candidates. Looking ahead, the application of deep learning methods could further improve prediction accuracy, though the limited availability of experimental data for model training presents a challenge for future developments.

## Supplementary Material

gkaf390_Supplemental_File

## Data Availability

SIREs 3.0 web server is freely available to all users at https://www.sires-webserver.eu/. Data will be made available by the authors to other investigators upon reasonable request and addressed to the corresponding author. Scripts used in the case study have been deposited at https://zenodo.org/records/14849946.
