# Supplementary material for: SIREs 3.0, an improved RNA prediction tool for iron-responsive elements
Source: Nucleic Acids Res. 2025 May 5;53(W1):W520–7. doi: 10.1093/nar/gkaf390 (PMC12230718; doi:10.1093/nar/gkaf390)
Supplement: gkaf390_Supplemental_File [file gkaf390_supplemental_file.pdf]

Supplementary data

List of contents

- Benchmarking analysis
  - IREs predictors analyzed
  - Modified RNAmotif script to account for canonical IRE motifs: 1 & 2
  - Metrics for performance assessment
- Case study
  - Download and preprocessing of the input data
  - Processing of SIREs results
  - Obtention of non-redundant IREs
  - Orthology analysis
  - Calculation of IRE density per transcript region
- References
- Supplementary Figures
  - *Supplementary Figure 1.* Improved IRE scoring system in SIREs v3.0.
  - *Supplementary Figure 2.* Graphical output for IRE scoring and free energy predictions.
  - *Supplementary Figure 3.* Overview of the SIREs 3.0 web interface, highlighting key features.
  - *Supplementary Figure 4.* Distribution and density of predicted IREs across transcript regions in human and mouse.
- Supplementary Tables
  - *Supplementary Table 1.* Sequences used for gold-standard values calculation.
  - *Supplementary Table 2.* Sequences used for benchmarking.
  - *Supplementary Table 3.* Performance assessment of SIREs and two additional IREs predictive softwares without pooling adjacent categories.

- *Supplementary Table 4.* Predicted IREs in human genes and their mouse orthologs with the same motif and transcript location.
- *Supplementary Table 5.* Detection of literature-reported IREs by SIREs 3.0, RNAAnalyzer and RNAMotif.

Benchmarking analysis

IREs predictors analyzed

RNAAnalyzer.

In October 2024, we used the RNAAnalyzer web server (https://rnaanalyzer.bioapps.biozentrum.uni-wuerzburg.de) to analyze the positive and negative control sequences listed in Supplementary Table 2. This tool generates results in .html format, including a section that identifies the presence of IREs (Special RNA Structure Information > Iron-resp Ele.). In addition to IRE detection, the server provides secondary structure predictions and free energy values. From the generated results, we extracted the information regarding the presence or absence of IREs, based on the predicted positions of the IREs or the absence thereof (noted as "None").

RNAMotif.

We installed RNAMotif 3.1.1. on Ubuntu 24.04 in October 2024 (https://github.com/dacase/rnamotif) and modified the original script to allow identification of both motif 1 and motif 2 IREs. The original script, as described in the RNAMotif article [1], was designed to detect only motif 1 IREs. Using this modified version, we analyzed the same positive and negative control sequences from Supplementary Table 2. The program produces a tabular output, displaying the location, length, and sequence of each predicted motif. We reviewed these results to determine the presence or absence of IREs in the input sequences.

SIREs 3.0.

In December 2024, we used SIREs (https://www.sires-webserver.eu) in batch mode to analyze the positive and negative control sequences from Supplementary Table 2. We downloaded the corresponding GFF file summarizing the results and categorized the predicted IREs based on quality (High, High-Medium, Medium, Medium-Low, Low, and Very Low).

Modified RNAMotif script to account for canonical IRE motifs: 1 & 2

```
parms
wc += gu;
descr
        ss( len=3 )
        h5( tag="lower_stem", len=3 )
        ss( tag="5p_bulge", minlen=1, maxlen=3, tag="5p", seq="c$" )
        h5( tag="upper_stem", len=5 )
        ss( len=6, seq="^cag[ua]g*" )
        h3( tag="upper_stem")
        ss( tag="3p_bulge", minlen=0, maxlen=1)
        h3( tag="lower_stem")
        ss( len=3 )

        score
        {
        SCORE = 0.0;
        if( length(ss(tag="5p")) == 3 && length( ss( tag="3p_bulge")) == 1 ) {
        if( ss(tag="5p") == "tgc" )
        SCORE = 0.5;
        else if( ss(tag="5p") == "tac" )
        SCORE = 0.4;
        else if( ss(tag="5p") == "tcc" )
        SCORE = 0.3;
        else if( ss(tag="5p") == "ttc" )
        SCORE = 0.2;
        else if( ss(tag="5p",pos=1,len=1) == "c" )
        SCORE += 0.5;
        else if( ss(tag="3p_bulge") == "t" )
        SCORE += 0.2;
        ACCEPT;
        }else if( length(ss(tag="5p" )) == 1 && length(ss(tag="3p_bulge")) == 0 ){
        SCORE = 1.0;
        ACCEPT;
        }else
        REJECT;
        }
```

Metrics for performance assessment

To evaluate the performance of SIREs and the other two programs, we chose the following metrics.

**Sensitivity**	measures the percentage of real positives that the model correctly identifies	TP/(TP+FN)
**Specificity**	ability to correctly identify true negatives, meaning cases where no IRE is detected.	TN/(TN+FP)
**Balanced Accuracy**	average of sensitivity and specificity, giving a more balanced view of model performance.	(Sensitivity+Specificity)/2
**Precision**	measures how many of the predicted positives were real positives.	TP/(TP+FP)

Case study

Download and preprocessing of the input data

We downloaded RefSeq data for both human and mouse genomes, using the following assemblies:

- Human: GRCh38.p14 (the latest primary assembly, released in 2013)
- Mouse: GRCm39 (the latest primary assembly, released in 2020)

For both species, we focused on transcripts annotated with the NM_ identifier, which indicates mRNA sequences. After obtaining the data, we processed it to count the sequences per file and filtered out only those with the NM_ annotation.

The filtered datasets were then split into SIREs-friendly files, each approximately 3Mb in size (containing roughly 800 sequences). These smaller files were submitted to the SIREs web server using batch mode for IRE prediction.

Processing of SIREs results

Following the batch analysis, we retrieved the GFF files generated by SIREs for further examination. Custom in-house R scripts were developed to preprocess these files, removing unnecessary data while retaining relevant information for downstream analysis. Additionally, we implemented scripts to extract transcript lengths for those predicted to harbor an IRE, as well as the lengths of transcript regions, including the 5' UTR, CDS, and 3' UTR.

Obtention of non-redundant IREs

Since many genes have multiple transcripts, we aimed to eliminate redundancy by identifying non-redundant IREs. We defined redundant IREs as those that, despite being found in different transcripts of the same gene, shared the same motif, mismatch, bulge, N25 sequence, number of wobble pairs in the upper stem, and canonical energy. Only non-redundant IREs were retained for subsequent analyses, including orthology comparisons and IRE density calculations.

Orthology analysis

Mouse orthologs of human genes were identified using bioMART 2.60.1 [2], restricting our analysis to one-to-one orthology relationships to ensure consistency and comparability between species.

Calculation of IRE density per transcript region

To assess IRE distribution across transcript regions, we calculated the average lengths of the 5’ UTR, CDS, and 3’ UTR from the transcripts included in our study. We then normalized the total IRE counts by region length, yielding a density value that facilitates comparisons and enhances the interpretation of IRE enrichment in different transcript regions.

Scripts to produce the manuscript figures and reproduce the case study analyses have been deposited at:

References

1. Macke TJ, Ecker DJ, Gutell RR, Gautheret D, Case DA, Sampath R. RNAMotif, an RNA secondary structure definition and search algorithm. Nucleic Acids Res [Internet]. 2001 Nov 15;29(22):4724–35. Available from: http://dx.doi.org/10.1093/nar/29.22.4724
2. Durinck S, Spellman P, Birney E, Huber W (2009). “Mapping identifiers for the integration of genomic datasets with the R/Bioconductor package biomaRt.” Nature Protocols, 4, 1184–1191.

Supplementary Figures

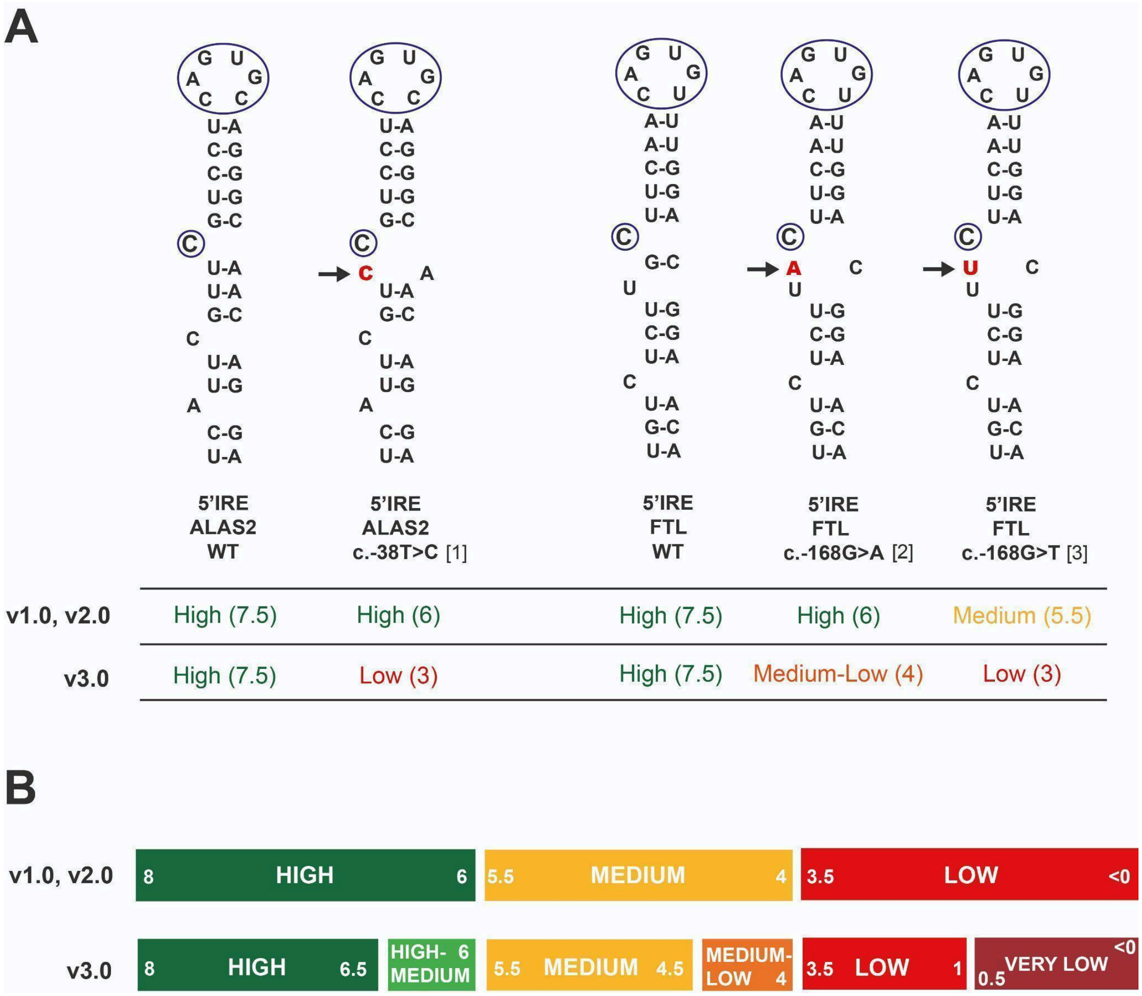

Supplementary Figure 1. Improved scoring system in SIREs v3.0. (A) Comparison of IRE scoring for *ALAS2* and *FTL* across different versions of the SIREs program. On the left, the wild-type (WT) IRE of *ALAS2* is shown alongside its mutated form (c.-38T>C [1]), with a table below displaying the scoring results from SIREs v1.0 and v2.0, and v3.0. In v3.0, the improved scoring system correctly classifies the mutated IRE as a low-quality prediction, rather than maintaining a "high" quality score. On the right, a similar analysis is shown for *FTL*, including the WT IRE and two mutations (c.-168G>A [2] and c.-168G>U [3]), where the updated scoring system appropriately adjusts their classification to lower categories. (B) Expansion of the IRE scoring scale in SIREs version 3. The previous three-category system (High, Medium, Low) used in versions 1 and 2 has been refined into a six-category system (High, High-Medium, Medium, Medium-Low, Low, Very Low), allowing for more precise differentiation of IRE quality predictions.

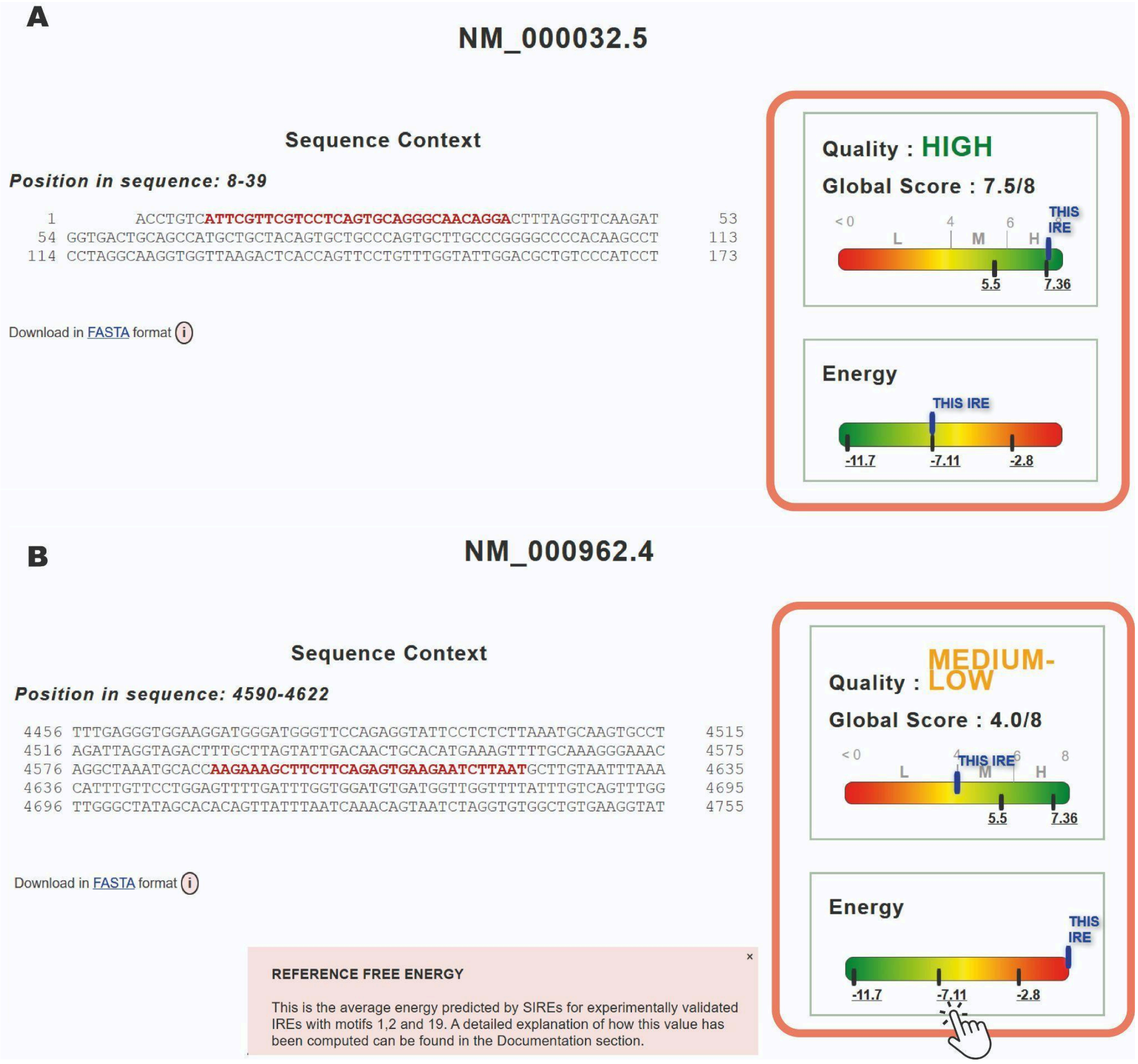

Supplementary Figure 2. Graphical output for IRE scoring and free energy predictions. (A) Example of the graphical output for the prediction of *ALAS2* transcript NM_00032.5. The top box displays a scoring bar ranging from <0 to 8, where the predicted IRE is classified as HIGH quality. The bottom box presents a free energy bar, spanning from a negative value to 0, where the predicted free energy is close to the reference average (-7.11 kcal/mol). (B) Graphical output for the prediction of *PTGS1* transcript NM_000962.4. The scoring bar classifies this IRE as medium-low, while the free energy prediction is 0.0, significantly deviating from gold-standard IREs. These graphical outputs provide context by visually comparing the predicted values against validated IRE references. The reference values, displayed at the bottom of each bar, can be clicked to open a pop-up explaining how they were computed. An example is shown for the reference free energy value of -7.11 kcal/mol.

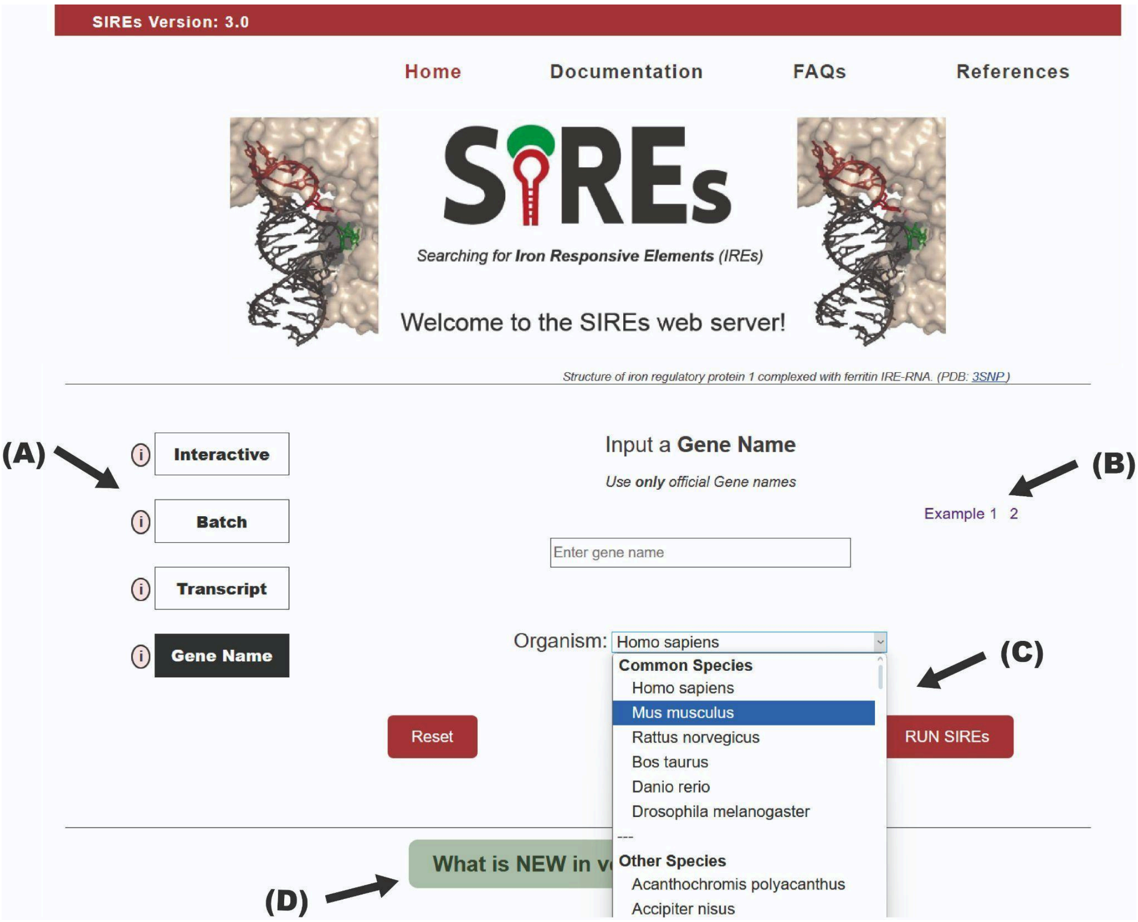

Supplementary Figure 3. Overview of the SIREs 3.0 web interface, highlighting key features. (A) New buttons to switch between SIREs input modes, each with an adjacent help pop-up for quick submission guidance. (B) Preloaded example inputs to illustrate the expected format for SIREs submissions. (C) Close-up of the dropdown menu in Gene mode, allowing users to select species for analysis, with commonly used species listed separately for convenience (list adapted from ENSEMBL). (D) A dedicated "What's New in Version 3.0" page summarizing all updates in SIREs 3.0 and improvements introduced since SIREs 1.0, accessible at this link.

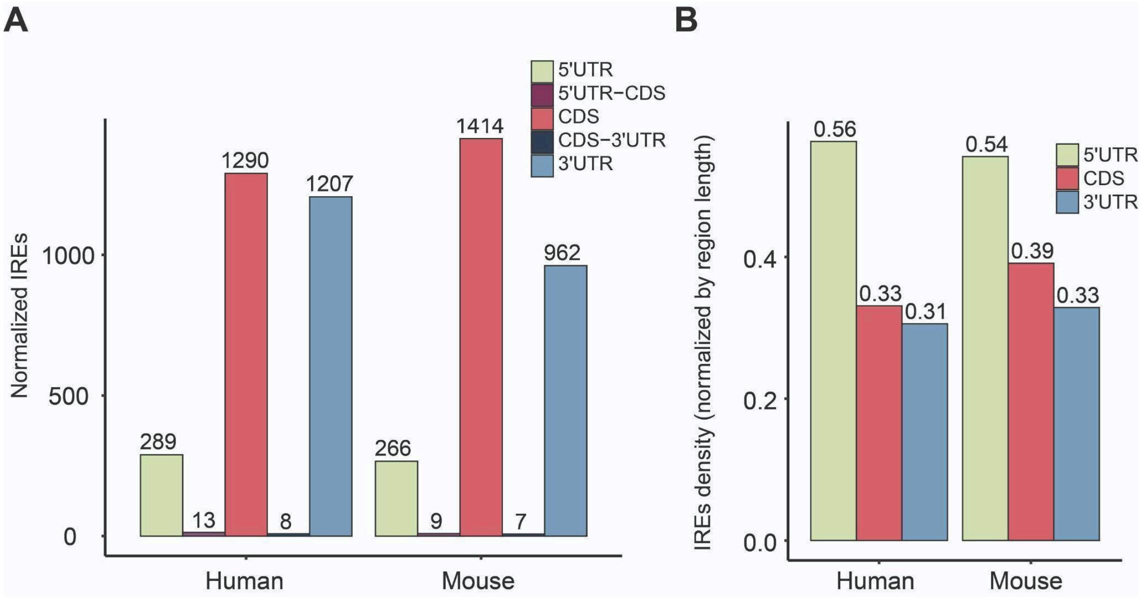

Supplementary Figure 4. Distribution and density of predicted IREs across transcript regions in human and mouse. (A) Bar plot showing the total counts of predicted IREs across different transcript regions (5’UTR, 5’UTR-CDS, CDS, CDS-3’UTR, and 3’UTR) in human and mouse transcripts. The majority of predicted IREs are found within the CDS (45.96% and 61.91%, respectively) and 3’UTR (42.99% and 40.40%, respectively), while only a small fraction is located in the 5’UTR (10.30% and 11.17%, respectively), and even fewer in the 5’UTR-CDS (0.46% and 0.38%, respectively) and CDS-3’UTR (0.28% and 0.29%, respectively) regions. (B) Bar plot showing the normalized IRE density across major transcript regions (5’UTR, CDS, and 3’UTR). When adjusted for region length, the 5’UTR shows the highest relative occurrence of IREs in human and mouse (0.56 and 0.54, respectively). In contrast, the CDS and 3’UTR show lower enrichment, despite harbouring a larger absolute number of IREs.

Supplementary Tables

Supplementary Table 1. Sequences used for gold-standard values calculation.

Motif	Transcript_Gene	Score	Canonical Energy	
19	NM_019410.3_Pfn2	7	-10.7	
19	NM_053024.4_PFN2	7	-8.3	
1	NM_010240.2_Ftl1	8	-6.7	
1	NM_000146.4_FTL	7.5	-5.3	
2	NM_011638.4_Tfrc	8	-7.2	IRE A
1	NM_011638.4_Tfrc	8	-8.4	IRE B
1	NM_011638.4_Tfrc	7.5	-9.2	IRE C
1	NM_011638.4_Tfrc	8	-9.7	IRE D
1	NM_011638.4_Tfrc	8	-11.7	IRE E
2	NM_001128148.3_TFRC	8	-6.9	IRE A
1	NM_001128148.3_TFRC	8	-8.4	IRE B
1	NM_001128148.3_TFRC	7.5	-9.8	IRE C
1	NM_001128148.3_TFRC	8	-9	IRE D
1	NM_001128148.3_TFRC	8	-11.7	IRE E
1	**NM_016917.2_Slc40a1**	8	**-10.8**	
1	**NM_014585.6_SLC40A1**	8	**-10.8**	
1	**NM_080633.2_Aco2**	6	**-2.8**	
1	**NM_001098.3_ACO2**	6	**-2.8**	
1	**ENSMUST00000138843_Slc11a2**	**5.5**	**-7.1**	
1	**NM_001174129.2_SLC11A2**	**5.5**	**-6.3**	
1	AK158430.1_Epas1	6	-4.7	
1	NM_001430.5_EPAS1	6	-4.7	
1	NM_009653.4_Alas2	7.5	-6.9	
1	NM_000032.5_ALAS2	7.5	-7	
1	NM_010239.2_Fth1	8	-7.2	
1	NM_002032.3_FTH1	7.5	-6.7	
1	NM_003672.4_CDC14A	8	-5	
1	FBtr0086156_SdhB	8	-6.4	

Supplementary Table 2. Sequences used for benchmarking.

Positive controls	Negative controls
NM_010240.2_Ftl1	NM_011434.2
NM_000146.4_FTL	NM_001291022.2
NM_011638.4_Tfrc	NM_172839.4
NM_001128148.3_TFRC	NM_001362311.1
NM_016917.2_Slc40a1	NM_001416137.1
NM_014585.6_SLC40A1	NM_001411575.1
NM_080633.2_Aco2	NM_207214.3
NM_001098.3_ACO2	NM_025345.2
NM_008049.2_Ftl2	NM_001301157.1
NM_001356952.1_Slc11a2	NM_001368235.1
NM_001174129.2_SLC11A2	NM_001424794.1
AK158430.1_Epas1	NM_001253752.1
NM_001430.5_EPAS1	NM_001404648.1
NM_009653.4_Alas2	NM_001426040.1
NM_000032.5_ALAS2	NM_146763.2
NM_010239.2_Fth1	NM_001420445.1
NM_002032.3_FTH1	NM_001081436.3
NM_019410.3_Pfn2	NM_021452.2
NM_002628.5_PFN2	NM_001362525.1
NM_001319210.2_CDC14A	NM_001412958.1
NM_177088.3_Cep95	NM_001302336.2
NM_008761.5_Fxyd5	NM_028364.3
NM_145517.4_Ormdl1	NM_181589.3
NM_178111.4_Trp53inp2	NM_174854.2
NM_018810.3_Mkrn1	NM_001355561.1
NM_026850.4_Pdcl3	NM_001374200.1
NM_029802.4_Arfip2	NM_001419593.1
AK157080.1_D5Ertd255e	NM_001313918.1
NM_010727.4_Lnx1	NM_001414220.1
NM_172947.3_Lsm12	NM_001316367.1
NM_001190371.1_Ankrd29	NM_009747.2
NM_015743.4__Nr4a3	NM_008379.3
NM_001411621.1_Pyroxd1	NM_001426778.1
NM_146014.3_Ccm2	NM_001403437.2

NM_027854.1_Tmem248	NM_001286750.1
NM_013587.3_Lrpap1	NM_001411964.1
NM_023158.7_Cxcl16	NM_001285937.1
NM_013755.5_Gyg1	NM_008902.3
NM_019994.5_Ralgapa1	NM_001426345.1
NM_010118.3_Egr2	NM_010672.3
NM_001081436.3_Ino80d	NM_001358520.1
NM_173737.3_Hmces	NM_001410572.1
NM_201531.5_Kcnf1	NM_001162524.2
NM_010403.2_Hao1	NM_175270.5
NM_018810.3_Mkrn1	NM_001373885.1
NM_008184.4_Gstm6	NM_001420992.1
AK134743.1_AI450353	NM_001033284.3
NM_183170.2_Mpv17l2	NM_001418869.1
NM_144828.2_Ppp1r1b	NM_144547.2
NM_133994.3_Gstt3	NM_009460.2
NM_011807.3_Dlg2	NM_172643.6
NM_001101479.2_Pabpc4l	NM_027271.1
NM_178629.6_Crppa	NM_029457.3
NM_133941.3_Dhx32	NM_001378663.1
NM_153550.4_Slc49a4	NM_001385627.1
NM_001025573.2_Slc23a4	NM_001426075.1
	NM_001011524.1
	NM_026429.4
	NM_001085502.2
	NM_033571.3
	NM_001416610.1
	NM_025377.3
	NM_001428460.1
	NM_001379684.1
	NM_001369356.1
	NM_001039073.2
	NM_001403900.1
	NM_001355396.1
	NM_001385189.1
	NM_025468.2
	NM_172253.2

	NM_001081066.2
	NM_147089.2
	NM_028226.2
	NM_001412466.1
	NM_021362.2
	NM_001356452.1
	NM_013807.3
	NM_013479.2
	NM_001159534.2
	NM_007627.6
	NM_001402839.1
	NM_001001804.2
	NM_008497.2
	NM_001409923.1
	NM_001425038.1
	NM_001428878.1
	NM_175283.3
	NM_001425805.1
	NM_001410302.1
	NM_178721.4
	NM_001110252.2
	NM_001348200.1
	NM_001145877.2
	NM_026776.5
	NM_010744.4
	NM_213660.3
	NM_001356967.1
	NM_001013580.4
	NM_001409463.1
	NM_001347477.1
	NM_001099792.1
	NM_001374758.1
	NM_146003.2
	NM_001370828.1
	NM_001409748.1
	NM_001422975.1
	NM_001286425.2

	NM_010024.3
	NM_001293806.1
	NM_021447.2
	NM_203280.3
	NM_009930.2
	NM_026638.4
	NM_001355212.2
	NM_001408599.1
	NM_001285886.1
	NM_001290563.1
	NM_001290637.1
	NM_146686.2
	NM_001253796.1
	NM_001417002.1
	NM_001277257.1
	NM_001403056.1
	NM_001414258.1
	NM_009736.3
	NM_001424687.1
	NM_001109993.1
	NM_001357424.1
	NM_001361144.1
	NM_001289622.1
	NM_001428692.1
	NM_001416278.1
	NM_001381953.1
	NM_001425744.1
	NM_001369274.1
	NM_146471.1
	NM_001134743.1
	NM_001359293.2
	NM_001164081.1
	NM_001110133.2
	NM_012049.3
	NM_001001452.1
	NM_028460.2
	NM_181075.3

	NM_027898.4
	NM_009471.3
	NM_013507.3
	NM_001017427.1
	NM_030207.3

Positive controls: in green, human and mouse transcripts that have validated IREs. In white, IRP-target mRNAs identified as binding both IRP1 and IRP2 in Sanchez et al., 2011.

Negative controls: mouse NM_ transcripts randomly selected from the NCBI database. Curated to avoid duplicated transcripts of the same gene.

References:

Sanchez,M., Galy,B., Schwanhaeusser,B., Blake,J., Bähr-Ivacevic,T., Benes,V., Selbach,M., Muckenthaler,M.U. and Hentze,M.W. (2011) Iron regulatory protein-1 and -2: transcriptome-wide definition of binding mRNAs and shaping of the cellular proteome by iron regulatory proteins. Blood, 118, e168–79.

Supplementary Table 3. Performance assessment of SIREs and two additional IREs predictive softwares without pooling adjacent categories

	**RNAMotif**	**RNAAnalyzer**	**SIREs 3.0** Quality sub-category			
			High	*High-Medium*	*Medium*	*Medium-Low*
Sensitivity	32.08	33.96	41.51	11.31	13.21	9.44
Specificity	97.33	98.00	98.67	100.00	91.33	98.00
Precision	80.95	85.71	91.67	100.00	35.00	62.50
Balanced Accuracy	65.7	65.98	70.09	55.66	70.94	53.72

Supplementary Table 4. Predicted IREs in human genes and their mouse orthologs with the same motif and transcript location.

Motif	Sequence position	HUMAN IREs									MOUSE IREs								
		Human Gene	Transcript	Start	End	Mismatch	Bulge	Canonical energy	Quality	Score	Mouse Gene	Transcript	Start	End	Mismatch	Bulge	Canonical energy	Quality	Score
1	5'UTR	*ALAS2*	NM_000032.5	8	39	-	-	-7	High	7.5	*Alas2*	NM_001409472.1	11	42	-	-	-6.9	High	7.5
1	5'UTR	*FTH1*	NM_002032.3	32	63	-	-	-6.7	High	7.5	*Fth1*	NM_010239.2	86	117	-	-	-7.2	High	8
1	5'UTR	*FTL*	NM_000146.4	26	57	-	-	-5.3	High	7.5	*Ftl1*	NM_010240.2	79	110	-	-	-6.7	High	8
1	5'UTR	*SLC40A1*	NM_014585.6	97	128	-	-	-10.8	High	8	*Slc40a1*	NM_016917.2	101	132	-	-	-10.8	High	8
1	CDS	*ABCC1*	NM_019862.3	3621	3652	N13-N20:T_T	-	-9.4	Medium	4.5	*Abcc1*	NM_008576.4	3674	3705	N13-N20:T_T	-	-14	High	7.5
1	CDS	*BAP1*	NM_004656.4	1547	1578	-	-	-5.9	High	8	*Bap1*	NM_027088.2	1967	1998	N12-N21:T_C	-	-3.5	Medium	4.5
1	CDS	*COL14A1*	NM_001384947.1	1483	1514	N11-N22:C_A	-	-1.8	Medium	5.5	*Col14a1*	NM_001368422.1	1481	1512	N11-N22:C_A	-	-1.8	High	7.5
1	CDS	*CSF1R*	NM_001288705.3	2250	2281	N13-N20:A_C	-	-2.6	Medium	5.5	*Csf1r*	NM_001037859.2	2399	2430	N13-N20:A_C	-	-1.7	Medium	4.5
1	CDS	*DRP2*	NM_001939.3	3340	3371	N12-N21:C_C	-	-0.2	Medium	4.5	*Drp2*	NM_001411869.1	3186	3217	N12-N21:C_C	-	-1.3	Medium	4.5
1	CDS	*E4F1*	NM_001288778.2	847	878	N09-N24:C_A	-	-2.9	Medium	5	*E4f1*	NM_001301784.1	1174	1205	-	-	-3.7	High-Medium	6
1	CDS	*FBXW4*	NM_022039.4	1223	1254	N11-N22:G_A	-	-1.4	Medium	4.5	*Fbxw4*	NM_013907.3	1145	1176	N11-N22:G_A	-	-1.4	Medium	4.5
1	CDS	*GAREM2*	NM_001191033.2	2206	2237	N13-N20:T_C	-	-4.3	Medium	5	*Garem2*	NM_001167879.1	768	799	N13-N20:C_C	-	-4	Medium	5
1	CDS	*HIVEP2*	NM_006734.4	4700	4731	-	-	-5.2	Medium	5	*Hivep2*	NM_010437.2	4779	4810	-	-	-7	Medium	4.5
1	CDS	*IKBKE*	NM_014002.4	602	633	N09-N24:T_C	-	-6.8	High	6.5	*Ikbke*	NM_019777.3	603	634	N09-N24:T_C	-	-6.8	High	6.5
1	CDS	*INPPL1*	NM_001567.4	1393	1424	N13-N20:T_C	-	-3.8	Medium	5.5	*Inppl1*	NM_001412589.1	1534	1565	N13-N20:T_C	-	-0.8	High	6.5
1	CDS	*IQSEC2*	NM_001410736.1	973	1004	N09-N24:C_A	-	-7.7	High	6.5	*Iqsec2*	NM_001114664.3	1006	1037	N09-N24:C_A	-	-7.7	High	6.5
1	CDS	*LIMS3*	NM_001426397.1	314	345	-	-	-4.5	Medium	5	*Lims1*	NM_001359115.1	232	263	N07-N25:C_A	-	-8.9	Medium	4.5
1	CDS	*MDN1*	NM_014611.3	16927	16958	-	-	-4.2	Medium	4.5	*Mdn1*	NM_001081392.1	9879	9910	N13-N20:G_G	-	-3.9	Medium	5.5
1	CDS	*MTMR4*	NM_004687.5	191	222	N11-N22:T_C	-	-3.6	Medium	5.5	*Mtmr4*	NM_001402585.1	269	300	N11-N22:T_C	-	-3.5	Medium	4.5

1	CDS	*NR2C2*	NM_001291694.2	1647	1678	N12-N21:C_C	-	-5.6	Medium	4.5	*Nr2c2*	NM_001410478.1	1705	1736	N12-N21:C_C	-	-5.5	Medium	5.5
1	CDS	*PLB1*	NM_001170585.2	3213	3244	N09-N24:T_C	-	-1.9	Medium	4.5	*Plb1*	NM_001081407.1	3506	3537	N09-N24:T_C	-	-1.9	Medium	4.5
1	CDS	*PLEKHA6*	NM_014935.5	1275	1306	N13-N20:G_G	-	-4.7	High-Medium	6	*Plekha6*	NM_001160268.1	1145	1176	N13-N20:G_G	-	-4.7	High	6.5
1	CDS	*PLXNB1*	NM_002673.6	3930	3962	-	N23b:C	-2.8	Medium	5	*Plxnb1*	NM_172775.2	1820	1851	N11-N22:G_G	-	-4.5	Medium	4.5
1	CDS	*POLR3B*	NM_018082.6	1542	1574	-	N22b:A	-3.5	Medium	4.5	*Polr3b*	NM_027423.2	1579	1611	-	N22b:A	-2.7	Medium	5
1	CDS	*PTPRM*	NM_002845.4	1108	1139	N10-N23:A_C	-	-2.2	Medium	4.5	*Ptprm*	NM_001357625.1	1925	1956	-	-	-2.5	Medium	4.5
1	CDS	*R3HDM2*	NM_001351206.2	1557	1588	N11-N22:C_T	-	-2.2	High	6.5	*R3hdm2*	NM_001168293.1	1572	1603	N11-N22:C_T	-	-1.3	High	6.5
1	CDS	*RNF216*	NM_207111.4	2060	2091	N12-N21:A_C	-	-1.3	High	6.5	*Rnf216*	NM_080561.4	1843	1874	N12-N21:A_C	-	-4.1	Medium	5.5
1	CDS	*SHKBP1*	NM_138392.4	1651	1682	-	-	-6	High-Medium	6	*Shkbp1*	NM_138676.2	1676	1707	-	-	-6.2	High-Medium	6
1	CDS	*SLC4A3*	NM_201574.3	931	963	-	N23b:G	-4.9	High-Medium	6	*Slc4a3*	NM_009208.3	1499	1530	N13-N20:A_C	-	-0.3	Medium	5.5
1	CDS	*SNRNP200*	NM_014014.5	6186	6217	N09-N24:A_C	-	-3.3	Medium	5	*Snrnp200*	NM_177214.5	6185	6216	N09-N24:A_C	-	-4	Medium	5.5
1	CDS	*SRGAP2B*	NM_001385226.1	956	988	-	N22b:T	0	Medium	5.5	*Srgap2*	NM_001081011.2	3168	3200	-	N22b:T	-4.6	Medium	5
1	CDS	*STIL*	NM_001377417.1	3619	3650	N12-N21:A_C	-	-1.4	Medium	5	*Stil*	NM_001304551.1	3069	3100	N12-N21:A_C	-	-1.3	Medium	4.5
1	CDS	*TCERG1*	NM_001040006.2	2926	2957	N13-N20:C_C	-	-3.3	High	7.5	*Tcerg1*	NM_001039474.1	3018	3049	N13-N20:C_C	-	-3.3	High	7.5
1	CDS	*TGM2*	NM_001323316.2	988	1019	N11-N22:G_A	-	-4.5	Medium	5.5	*Tgm2*	NM_009373.3	936	967	-	-	-10.6	High-Medium	6
1	CDS	*TRIO*	NM_007118.4	7331	7362	N13-N20:A_C	-	-9.4	Medium	4.5	*Trio*	NM_001081302.1	684	715	N09-N24:A_G	-	-4.4	Medium	5
1	CDS	*TTN*	NM_001256850.1	16421	16452	N09-N24:A_G	-	-1.2	Medium	4.5	*Ttn*	NM_001385708.1	41023	41054	N11-N22:G_A	-	-1.4	Medium	5.5
1	CDS	*ZAP70*	NM_001378594.1	233	264	N13-N20:C_C	-	-5.7	Medium	5.5	*Zap70*	NM_001289765.2	1597	1628	N11-N22:C_T	-	-5.8	Medium	5.5
1	CDS	*ZC3H7A*	NM_014153.4	611	643	-	N21b:C	-1.9	Medium	5.5	*Zc3h7a*	NM_145931.3	598	630	-	N21b:C	0	Medium	4.5
1	CDS	*ZHX1*	NM_001017926.3	536	567	N13-N20:C_A	-	-6.2	High	7.5	*Zhx1*	NM_001042438.2	855	886	-	-	-9.3	High	7.5
1	3'UTR	*DVL2*	NM_004422.3	2597	2628	N07-N25:T_T	-	-5.1	Medium	4.5	*Dvl2*	NM_007888.4	2574	2605	N07-N25:T_C	-	-5.1	Medium	4.5
1	3'UTR	*SLC11A2*	NM_001174125.2	1817	1849	-	N22b:T	-6.3	Medium	5.5	*Slc11a2*	NM_001146161.1	1849	1881	-	N22b:T	-7.1	Medium	5.5
1	3'UTR	*TFRC*	NM_001313966.2	3294	3325	-	-	-9.8	High	7.5	*Tfrc*	NM_011638.4	3829	3860	-	-	-11.7	High	8
2	CDS	*CACTIN*	NM_021231.2	427	458	N09-N24:A_A	-	-6.9	Medium	5.5	*Cactin*	NM_027381.2	573	604	N09-N24:A_A	-	-5.5	Medium	5.5

2	CDS	*CCDC61*	NM_001267723.2	265	296	-	-	-3.9	High-Medium	6	*Ccdc61*	NM_001033314.4	498	529	N13-N20:C_A	-	-4.3	Medium	5.5
2	CDS	*CDH20*	NM_031891.4	2236	2267	N13-N20:G_G	-	-2.2	Medium	4.5	*Cdh20*	NM_001420677.1	2270	2301	N13-N20:G_G	-	-3.7	Medium	5
2	CDS	*COG4*	NM_001195139.2	1458	1489	N10-N23:A_A	-	-1.3	Medium	5	*Cog4*	NM_133973.2	124	156	-	N20b:G	-5	Medium	4.5
2	CDS	*IGSF1*	NM_001170962.2	2033	2065	-	N22b:T	-10.6	High-Medium	6	*Igsf1*	NM_177591.4	2025	2057	-	N22b:T	-10.6	High-Medium	6
2	CDS	*KAT6A*	NM_001305878.2	1578	1610	-	N22b:C	0	Medium	4.5	*Kat6a*	NM_001081149.2	1650	1682	-	N22b:C	-0.3	Medium	5.5
2	CDS	*KCNAB1*	NM_172160.3	838	870	-	N20b:A	-3.8	Medium	5	*Kcnab1*	NM_010597.6	1490	1521	-	-	-6.8	Medium	5
2	CDS	*KRT27*	NM_181537.4	1042	1073	N13-N20:A_C	-	-5.4	Medium	5	*Krt27*	NM_010666.2	555	586	N11-N22:C_C	-	-4.3	Medium	4.5
2	CDS	*MAP4K4*	NM_001242560.2	2120	2151	N13-N20:T_T	-	-4.3	Medium	4.5	*Map4k4*	NM_001252200.1	2222	2253	N13-N20:T_T	-	-7.3	Medium	4.5
2	CDS	*MORN4*	NM_001098831.2	572	603	N12-N21:C_C	-	-4.2	High	7	*Morn4*	NM_198108.2	597	628	N12-N21:C_C	-	-4.5	Medium	4.5
2	CDS	*PDE4DIP*	NM_001350520.2	5462	5493	-	-	-2.9	Medium	5	*Pde4dip*	NM_001039376.2	4969	5000	N13-N20:C_T	-	-1.5	Medium	4.5
2	CDS	*PKP3*	NM_001303029.2	1479	1511	-	N22b:T	-0.6	High-Medium	6	*Pkp3*	NM_019762.2	1503	1535	-	N22b:T	-0.6	High	6.5
2	CDS	*PRPF40B*	NM_001379032.1	799	830	N12-N21:A_G	-	-2.1	Medium	4.5	*Prpf40b*	NM_001348256.1	846	877	N12-N21:A_A	-	-2.2	Medium	5.5
2	CDS	*PSAPL1*	NM_001085382.2	1192	1223	-	-	-11	High	7	*Psapl1*	NM_175249.4	153	184	N11-N22:T_T	-	-2	Medium	4.5
2	CDS	*RAD21L1*	NM_001384358.1	1342	1373	N09-N24:T_C	-	-4.1	Medium	5.5	*Rad21l*	NM_001276400.1	1587	1618	N09-N24:T_C	-	-1.7	Medium	5
2	CDS	*SIPA1L3*	NM_015073.3	1972	2003	N11-N22:A_G	-	-4.2	Medium	4.5	*Sipa1l3*	NM_001081028.1	1231	1262	N09-N24:C_T	-	-5	High	7
2	CDS	*SKIDA1*	NM_207371.4	2596	2627	N10-N23:C_C	-	-10.7	Medium	4.5	*Skida1*	NM_028317.4	586	617	N10-N23:C_C	-	-10.7	Medium	4.5
2	CDS	*SPTBN4*	NM_020971.3	6521	6553	-	N23b:A	-7.8	Medium	5	*Sptbn4*	NM_001199234.1	2334	2366	-	N23b:A	-5.1	Medium	5
2	CDS	*SQLE*	NM_003129.4	1602	1634	-	N22b:A	-2.5	Medium	5.5	*Sqle*	NM_009270.3	1450	1482	-	N22b:A	-1	Medium	5
2	CDS	*TKT*	NM_001064.4	632	664	-	N20b:A	-5.8	Medium	4.5	*Tkt*	NM_009388.6	695	727	-	N20b:A	-6.8	High-Medium	6
2	CDS	*TMEM131L*	NM_015196.4	260	291	N09-N24:A_C	-	-1.2	High-Medium	6	*Tmem131l*	NM_001399526.1	261	292	N09-N24:A_C	-	-1.3	High	7.5
2	CDS	*TMEM245*	NM_032012.4	824	855	N12-N21:G_A	-	-5.2	Medium	4.5	*Tmem245*	NM_175518.5	61	92	N13-N20:C_C	-	-7.7	Medium	5.5
2	CDS	*TRA2A*	NM_001362759.2	177	208	N13-N20:G_G	-	-4	Medium	4.5	*Tra2a*	NM_001347209.2	180	211	N13-N20:G_G	-	-4	Medium	4.5
2	CDS	*TUBA4A*	NM_001278552.2	1496	1527	-	-	-6.1	High	7.5	*Tuba4a*	NM_001313723.2	766	797	N09-N24:A_C	-	-2.5	Medium	5

2	3'UTR	*CDK6*	NM_001145306.2	7804	7835	N10-N23:A_C	-	-2.1	Medium	4.5	*Cdk6*	NM_009873.3	6920	6951	N10-N23:C_C	-	0	Medium	5
2	3'UTR	*DENND6B*	NM_001001794.4	4577	4608	N07-N25:C_C	-	-7	Medium	4.5	*Dennd6b*	NM_027081.3	2208	2239	N11-N22:G_A	-	-9.7	Medium	4.5
2	3'UTR	*GRIK3*	NM_000831.4	5562	5593	N10-N23:G_G	-	-2.8	Medium	4.5	*Grik3*	NM_001081097.3	8652	8683	N12-N21:A_C	-	-0.7	Medium	4.5
2	3'UTR	*KCNB1*	NM_004975.4	5612	5643	N13-N20:C_C	-	-4.4	Medium	5.5	*Kcnb1*	NM_008420.4	7348	7379	N11-N22:A_G	-	-2.3	Medium	4.5
2	3'UTR	*KCNJ6*	NM_002240.5	6691	6722	N07-N25:G_A	-	-3.9	Medium	4.5	*Kcnj6*	NM_001025584.2	2263	2294	N09-N24:C_T	-	-6	Medium	4.5
2	3'UTR	*KDM2A*	NM_001256405.2	4440	4471	N09-N24:C_A	-	-5.3	High	7	*Kdm2a*	NM_001001984.2	6357	6388	N09-N24:C_A	-	-2.6	Medium	4.5
2	3'UTR	*SIPA1L3*	NM_015073.3	6345	6376	N09-N24:A_A	-	-4.9	Medium	5.5	*Sipa1l3*	NM_001081028.1	6584	6615	-	-	-10.8	High	6.5
3	5'UTR	*PALMD*	NM_017734.5	13	44	N10-N23:T_T	-	-4.3	Medium	4.5	*Palmd*	NM_023245.3	213	244	-	-	-4.1	Medium	4.5
3	CDS	*ABCA5*	NM_018672.5	5168	5199	N10-N23:C_A	-	-0.1	Medium	5	*Abca5*	NM_147219.2	4200	4231	N10-N23:C_A	-	-0.1	Medium	5
3	CDS	*ADCY8*	NM_001115.3	2714	2745	N10-N23:T_T	-	0	Medium	4.5	*Adcy8*	NM_001331075.1	3125	3156	N10-N23:T_T	-	0	Medium	4.5
3	CDS	*ARHGEF10L*	NM_001319838.1	767	799	-	N22b:A	-4	Medium	5	*Arhgef10l*	NM_001112722.1	1672	1704	-	N22b:A	-5.6	Medium	4.5
3	CDS	*CDH6*	NM_001362435.2	2080	2111	N11-N22:G_G	-	-0.5	Medium	4.5	*Cdh6*	NM_007666.4	2046	2077	N11-N22:G_G	-	-0.5	Medium	4.5
3	CDS	*CRY2*	NM_021117.5	1575	1606	N10-N23:C_A	-	-2.6	Medium	5	*Cry2*	NM_009963.4	1602	1633	N10-N23:C_A	-	-2.6	Medium	5
3	CDS	*KCND1*	NM_004979.6	2523	2554	N09-N24:A_G	-	-3.5	Medium	4.5	*Kcnd1*	NM_008423.2	1034	1065	N09-N24:A_G	-	-3.3	Medium	4.5
3	CDS	*NRG3*	NM_001165973.2	729	760	-	-	-6.7	High-Medium	6	*Nrg3*	NM_001190188.1	1412	1443	-	-	-6.7	High-Medium	6
3	CDS	*PYGM*	NM_001164716.1	2023	2054	N09-N24:T_C	-	-9.1	High	6.5	*Pygm*	NM_011224.2	1541	1572	N09-N24:T_C	-	-6.2	High-Medium	6
3	3'UTR	*DVL1*	NM_004421.3	3166	3197	N11-N22:C_A	-	-2.4	Medium	4.5	*Dvl1*	NM_001356381.1	3318	3349	N11-N22:C_A	-	-2.1	Medium	4.5
3	3'UTR	*PRKG1*	NM_001374781.1	5121	5152	N13-N20:G_A	-	-3.3	High	6.5	*Prkg1*	NM_011160.3	6095	6126	N13-N20:G_G	-	-3.7	High	6.5
3	3'UTR	*TNRC18*	NM_001080495.3	10794	10825	N10-N23:A_C	-	0	Medium	4.5	*Tnrc18*	NM_001122730.3	10494	10525	N10-N23:A_C	-	0	Medium	4.5
4	CDS	*NEFL*	NM_006158.5	239	270	N11-N22:T_C	-	-5.2	High-Medium	6	*Nefl*	NM_010910.2	242	273	N11-N22:T_C	-	-5.2	High-Medium	6
5	5'UTR	*RARG*	NM_001243731.2	71	102	-	-	-8.1	Medium	4.5	*Rarg*	NM_001411714.1	50	81	-	-	-8	Medium	4.5
5	CDS	*ATG16L2*	NM_001318766.2	1705	1736	N13-N20:C_C	-	0	Medium	4.5	*Atg16l2*	NM_001111111.1	1623	1654	N13-N20:C_C	-	0	Medium	4.5
5	CDS	*MYCL*	NM_001033081.3	946	977	N09-N24:C_A	-	-8.3	High-Medium	6	*Mycl*	NM_001303121.2	670	701	N09-N24:C_C	-	-8.3	High-Medium	6

5	CDS	*TMC7*	NM_001300732.2	1119	1150	N11-N22:G_A	-	-0.9	Medium	4.5	*Tmc7*	NM_172476.5	1044	1075	N11-N22:G_A	-	-0.9	Medium	4.5
6	CDS	*PWP1*	NM_001317963.2	1293	1325	-	N20b:G	-1.7	High-Medium	6	*Pwp1*	NM_133993.3	1273	1305	-	N20b:G	-1.8	Medium	4.5
6	CDS	*STAG2*	NM_001282418.2	988	1019	N10-N23:A_A	-	0	Medium	5.5	*Stag2*	NM_021465.4	926	957	N10-N23:A_A	-	0	Medium	5.5
6	3'UTR	*PBX1*	NM_002585.4	2416	2447	N10-N23:A_A	-	-2.1	High	6.5	*Pbx1*	NM_001291508.1	2331	2362	N10-N23:A_A	-	-1.2	High	6.5
6	3'UTR	*TEAD1*	NM_021961.6	6851	6882	-	-	-0.6	Medium	5	*Tead1*	NM_001166584.2	7282	7313	-	-	-0.5	Medium	5
7	CDS	*PEAR1*	NM_001353682.2	1732	1763	-	-	-7	Medium	5	*Pear1*	NM_001032413.1	1664	1695	-	-	-4.7	Medium	5
7	CDS	*RALGAPA2*	NM_020343.4	4372	4403	N13-N20:A_C	-	-2.3	Medium	5.5	*Ralgapa2*	NM_001408200.1	4624	4655	N13-N20:A_C	-	-2.3	Medium	5.5
7	CDS	*WASF1*	NM_001024935.2	1193	1224	N13-N20:C_A	-	-7.8	Medium	4.5	*Wasf1*	NM_031877.3	939	970	N13-N20:C_A	-	-10.8	Medium	4.5
8	5'UTR	*MFSD6*	NM_001375988.1	88	119	-	-	-12.3	High-Medium	6	*Mfsd6*	NM_133829.3	264	295	-	-	-9.3	Medium	5
8	CDS	*BTBD1*	NM_001011885.2	1221	1252	N13-N20:T_C	-	-0.1	High	6.5	*Btbd1*	NM_146193.2	1266	1297	N13-N20:T_C	-	-0.1	High	6.5
8	CDS	*FBXW9*	NM_032301.3	584	616	-	N22b:T	-1.9	Medium	4.5	*Fbxw9*	NM_026791.2	543	575	-	N22b:G	-0.9	Medium	5.5
8	CDS	*HIC1*	NM_001098202.1	2127	2158	-	-	-9.3	Medium	5	*Hic1*	NM_001098203.1	2349	2380	N11-N22:A_G	-	-3.4	Medium	4.5
8	CDS	*MBP*	NM_002385.3	181	213	-	N20b:G	-3.2	Medium	4.5	*Mbp*	NM_001025245.1	824	856	-	N20b:G	-3.2	Medium	4.5
8	CDS	*TTLL4*	NM_014640.5	972	1003	N09-N24:C_C	-	-0.9	Medium	4.5	*Ttll4*	NM_001014974.2	1041	1073	-	N23b:T	-1.8	Medium	5.5
8	CDS	*ZFTA*	NM_001144936.2	182	213	N12-N21:G_G	-	-6.6	Medium	4.5	*Zfta*	NM_175381.7	192	223	N12-N21:G_G	-	-12.5	Medium	5.5
9a	3'UTR	*PCDH19*	NM_001184880.2	6614	6645	N12-N21:C_T	-	0	Medium	4.5	*Pcdh19*	NM_001105246.1	6561	6592	N12-N21:C_C	-	0	Medium	4.5
9b	5'UTR	*FIGN*	NM_018086.4	198	230	-	N20b:A	-0.5	Medium	5.5	*Fign*	NM_001267846.1	224	256	-	N20b:A	-0.5	Medium	5.5
9b	CDS	*PKHD1L1*	NM_177531.6	11668	11699	N09-N24:T_T	-	-1.8	Medium	4.5	*Pkhd1l1*	NM_138674.3	11645	11676	-	-	-1	Medium	5
11	CDS	*TEX15*	NM_001350162.2	6423	6455	-	N20b:T	0	Medium	4.5	*Tex15*	NM_031374.2	5113	5145	-	N23b:G	-0.5	Medium	5
13	3'UTR	*BCL11A*	NM_001405709.1	5612	5643	N09-N24:T_T	-	-1.3	High-Medium	6	*Bcl11a*	NM_001242934.1	5830	5861	N09-N24:T_T	-	-1.3	High-Medium	6
14	5'UTR	*CDK19*	NM_001300960.2	86	117	N09-N24:T_C	-	-11.5	Medium	4.5	*Cdk19*	NM_001291816.1	63	94	N09-N24:T_C	-	-7.8	Medium	4.5
14	CDS	*ADAM22*	NM_001324420.2	713	744	N11-N22:A_A	-	-1.5	High-Medium	6	*Adam22*	NM_001007220.4	672	703	N11-N22:A_A	-	-1.8	Medium	5
14	CDS	*ADAMTS2*	NM_021599.4	137	169	-	N20b:T	-12.4	Medium	5	*Adamts2*	NM_175643.3	83	115	-	N20b:T	-12.4	Medium	5
14	CDS	*FOXN3*	NM_005197.4	1453	1485	-	N21b:A	-9.7	Medium	4.5	*Foxn3*	NM_183186.2	1107	1139	-	N21b:A	-2	Medium	4.5

14	CDS	*PER1*	NM_002616.3	1538	1569	N11-N22:C_A	-	-4.1	Medium	4.5	*Per1*	NM_001159367.2	1508	1539	N11-N22:C_A	-	-4.9	Medium	4.5
14	CDS	*PPP1R3F*	NM_033215.5	989	1020	N09-N24:A_C	-	-7.8	High	6.5	*Ppp1r3f*	NM_001290574.2	963	994	N09-N24:A_C	-	-9.5	High-Medium	6
15	CDS	*UBR2*	NM_001363705.2	5300	5332	-	N20b:C	-7.4	Medium	5.5	*Ubr2*	NM_001177374.2	5322	5354	-	N20b:C	-7.5	Medium	5
16	5'UTR	*EED*	NM_001330334.2	250	281	-	-	-9.6	Medium	5	*Eed*	NM_021876.3	225	256	-	-	-8.3	Medium	4.5
16	CDS	*ATXN2L*	NM_001308230.2	1258	1289	N09-N24:A_C	-	-12	High	6.5	*Atxn2l*	NM_001361487.2	1469	1501	-	N23b:C	-10.5	Medium	5.5
16	CDS	*CAD*	NM_004341.5	5191	5223	-	N22b:T	-6.3	Medium	5	*Cad*	NM_001289523.2	5167	5199	-	N22b:T	-2.2	Medium	4.5
16	CDS	*CSGALNACT1*	NM_001354476.2	1311	1343	-	N23b:A	-8.3	High	6.5	*Csgalnact1*	NM_001252623.1	1866	1897	N09-N24:A_A	-	-3	Medium	4.5
16	CDS	*CSMD2*	NM_001281956.2	8823	8854	-	-	-11.8	High	7	*Csmd2*	NM_001281955.1	8656	8687	N13-N20:A_C	-	-12.9	High	6.5
16	CDS	*KCNK12*	NM_022055.2	1180	1211	N12-N21:C_C	-	-10	Medium	5.5	*Kcnk12*	NM_199251.1	1066	1097	N12-N21:C_C	-	-10	Medium	5.5
16	CDS	*MAPK8*	NM_001278547.2	826	858	-	N20b:A	-0.3	High-Medium	6	*Mapk8*	NM_001310452.2	820	852	-	N20b:A	-0.3	High-Medium	6
16	CDS	*MYOM2*	NM_003970.4	619	650	-	-	-7.7	High-Medium	6	*Myom2*	NM_001414182.1	1126	1157	-	-	-7.5	Medium	5.5
16	CDS	*RECK*	NM_021111.3	1660	1691	N11-N22:A_C	-	-0.7	Medium	5.5	*Reck*	NM_016678.3	1606	1637	N11-N22:A_C	-	-3.1	High-Medium	6
16	CDS	*SIL1*	NM_022464.5	898	929	N10-N23:A_G	-	-4.4	Medium	4.5	*Sil1*	NM_030749.2	944	975	N10-N23:A_G	-	-4.4	Medium	4.5
16	CDS	*SPATA2L*	NM_152339.4	857	888	-	-	-7.8	Medium	5	*Spata2l*	NM_001357300.1	1213	1244	-	-	-7.8	Medium	5
17	CDS	*RAD54L*	NM_001142548.2	1434	1465	-	-	-10.3	High	6.5	*Rad54l*	NM_001424848.1	916	947	-	-	-9.7	High	6.5
17	CDS	*RBCK1*	NM_006462.6	1196	1227	N13-N20:T_C	-	-8.9	High-Medium	6	*Rbck1*	NM_001421247.1	1233	1264	N13-N20:T_C	-	-8.9	High-Medium	6
17	CDS	*SETD2*	NM_001349370.3	4658	4690	-	N23b:C	-4.2	High-Medium	6	*Setd2*	NM_001081340.3	4547	4579	-	N23b:C	-4.2	High-Medium	6
18	5'UTR	*KCNJ15*	NM_170736.3	40	71	N13-N20:A_A	-	-6.8	Medium	5	*Kcnj15*	NM_001271691.1	207	238	N13-N20:A_A	-	-4.4	High	6.5
18	CDS	*EML3*	NM_001300794.2	949	980	-	-	-7	Medium	5.5	*Eml3*	NM_144872.1	871	902	-	-	-3.2	Medium	5
18	CDS	*LYSMD1*	NM_212551.5	732	763	N09-N24:C_A	-	-8.3	High	6.5	*Lysmd1*	NM_153121.2	817	848	N09-N24:C_A	-	-5.6	High	6.5
18	CDS	*TENM3*	NM_001415961.1	6236	6267	N09-N24:C_C	-	-2.1	Medium	5	*Tenm3*	NM_001145937.1	6707	6738	N09-N24:C_T	-	-4.6	High-Medium	6
18	CDS	*TIAM2*	NM_012454.4	1588	1620	-	N21b:A	-3.1	Medium	5	*Tiam2*	NM_001122998.1	4687	4719	-	N23b:G	-4.8	Medium	4.5
18	CDS	*TRAF4*	NM_004295.4	230	261	-	-	-6.9	Medium	5.5	*Traf4*	NM_009423.4	238	269	-	-	-4	Medium	5

18	3'UTR	*CDV3*	NM_001134422.2	1239	1270	N10-N23:A_A	-	-1.6	Medium	4.5	*Cdv3*	NM_001134427.1	1137	1168	N10-N23:A_G	-	-1.8	Medium	5
19	CDS	*ESRP1*	NM_001122825.2	1774	1805	N13-N20:T_C	-	-2.1	High	6.5	*Esrp1*	NM_194055.3	1976	2007	N13-N20:T_C	-	-2.1	High	6.5
19	CDS	*GLRA3*	NM_006529.4	1516	1547	N12-N21:A_A	-	-0.5	Medium	4.5	*Glra3*	NM_080438.4	1565	1596	N12-N21:A_A	-	-0.5	Medium	4.5
19	CDS	*SACS*	NM_014363.6	11617	11648	N13-N20:A_A	-	-1.6	Medium	4.5	*Sacs*	NM_015788.2	11809	11840	N13-N20:A_A	-	-1.6	Medium	4.5
19	CDS	*TBP*	NM_001172085.2	954	985	N12-N21:A_A	-	-0.4	Medium	4.5	*Tbp*	NM_013684.3	1091	1122	N12-N21:A_A	-	-0.4	Medium	4.5
19	CDS	*WDR62*	NM_173636.5	3234	3265	N09-N24:A_G	-	-7.4	Medium	5.5	*Wdr62*	NM_001408661.1	3285	3316	N09-N24:A_G	-	-5.3	Medium	5.5
19	3'UTR	*GFRA2*	NM_001495.5	2260	2291	N11-N22:G_G	-	-1	Medium	4.5	*Gfra2*	NM_001302094.1	2100	2132	-	N23b:A	-1	Medium	5
19	3'UTR	*HDAC9*	NM_001204146.2	4292	4323	N12-N21:T_T	-	0	Medium	5.5	*Hdac9*	NM_024124.3	4399	4430	N12-N21:T_T	-	0	Medium	5.5
19	3'UTR	*NEDD4*	NM_006154.4	5694	5725	N09-N24:A_C	-	0	Medium	5.5	*Nedd4*	NM_001357998.1	6349	6380	N09-N24:A_C	-	0	Medium	5.5
19	3'UTR	*PFN2*	NM_002628.5	693	724	-	-	-8.3	High	7	*Pfn2*	NM_019410.3	1027	1058	-	-	-10.7	High	7
20	CDS	*ADAMTSL1*	NM_001040272.6	1308	1339	N10-N23:A_A	-	-3.9	Medium	5	*Adamtsl1*	NM_029967.3	1320	1351	N10-N23:A_A	-	-3.9	Medium	5
20	CDS	*CSMD1*	NM_033225.6	11170	11201	-	-	-1.1	Medium	5.5	*Csmd1*	NM_053171.2	11083	11114	N09-N24:G_G	-	-1.6	Medium	5
20	CDS	*CYBRD1*	NM_001256909.2	359	390	N11-N22:G_G	-	-1.6	Medium	5.5	*Cybrd1*	NM_028593.2	559	590	N11-N22:G_G	-	-2.6	Medium	5.5
20	CDS	*DYNC2H1*	NM_001377.3	3957	3988	N11-N22:G_A	-	0	Medium	4.5	*Dync2h1*	NM_029851.3	3909	3940	N12-N21:A_C	-	-1.7	Medium	4.5
20	CDS	*GRIN2B*	NM_000834.5	2593	2624	N12-N21:G_G	-	-2.3	Medium	5.5	*Grin2b*	NM_001363750.1	2516	2547	N12-N21:G_G	-	-2.3	Medium	5.5
20	CDS	*ITGB3*	NM_000212.3	143	175	-	N23b:C	-10.9	Medium	4.5	*Itgb3*	NM_016780.2	194	226	-	N23b:C	-8.7	Medium	4.5
20	CDS	*LDB3*	NM_001080116.1	658	689	N10-N23:A_C	-	-4.1	Medium	4.5	*Ldb3*	NM_001039072.2	637	668	N10-N23:A_C	-	-6.9	Medium	4.5
20	CDS	*LRP1*	NM_002332.3	1216	1247	N12-N21:G_G	-	-4.9	Medium	5	*Lrp1*	NM_008512.2	1198	1229	N12-N21:G_G	-	-6.6	Medium	5.5
20	CDS	*MAGED2*	NM_201222.3	1317	1348	-	-	-5.8	High-Medium	6	*Maged2*	NM_030700.3	1284	1315	-	-	-5.8	High-Medium	6
20	CDS	*MAP7D2*	NM_001168465.2	2132	2163	-	-	-3.9	High-Medium	6	*Map7d2*	NM_001313751.1	2154	2185	-	-	-4.1	High-Medium	6
20	CDS	*MCM4*	NM_182746.3	2636	2667	N13-N20:A_C	-	-1.6	Medium	4.5	*Mcm4*	NM_008565.3	2765	2796	N13-N20:A_C	-	-2.9	Medium	5
20	CDS	*NBEA*	NM_015678.5	1717	1748	-	-	-3.7	High	6.5	*Nbea*	NM_030595.1	1602	1633	N10-N23:T_T	-	0	Medium	5
20	CDS	*NOMO1*	NM_014287.4	2632	2663	N10-N23:C_C	-	-7.4	High-Medium	6	*Nomo1*	NM_153057.4	2613	2644	N10-N23:C_T	-	-6.1	High-Medium	6

20	CDS	*PHLPP2*	NM_001289003.1	1267	1298	N09-N24:T_T	-	-2.9	High-Medium	6	*Phlpp2*	NM_001122594.2	653	684	N09-N24:T_T	-	-3.2	High-Medium	6
20	CDS	*PIK3C2G*	NM_001288772.2	4136	4168	-	N21b:G	-2	Medium	5	*Pik3c2g*	NM_011084.3	1535	1567	-	N21b:G	-2	Medium	4.5
20	CDS	*STIM1*	NM_001277961.3	1145	1176	N12-N21:T_C	-	-9.7	Medium	4.5	*Stim1*	NM_009287.5	1173	1204	-	-	-16.4	Medium	5
20	CDS	*TRAPPC8*	NM_014939.5	1751	1782	-	-	-7.9	High	6.5	*Trappc8*	NM_029491.2	1398	1429	N12-N21:A_A	-	-6.3	High	6.5
20	CDS	*TXLNG*	NM_018360.3	264	295	N09-N24:G_A	-	-1.1	Medium	5	*Txlng*	NM_178935.5	356	388	-	N23b:A	-1.1	Medium	5
20	CDS	*XPO5*	NM_020750.3	3383	3415	-	N20b:C	-4.9	Medium	5	*Xpo5*	NM_028198.3	3319	3351	-	N20b:C	-1.4	Medium	5
20	3'UTR	*BCAT1*	NM_001178091.2	2035	2066	N13-N20:A_A	-	-1.1	High-Medium	6	*Bcat1*	NM_001024468.3	6520	6552	-	N21b:C	-1.3	Medium	4.5
20	3'UTR	*ESRRA*	NM_001282450.2	1930	1961	N12-N21:G_A	-	-6.5	Medium	4.5	*Esrra*	NM_001413229.1	1442	1474	-	N23b:C	-8.1	High	6.5
20	3'UTR	*PCDH10*	NM_032961.3	8293	8324	N12-N21:T_T	-	-0.5	Medium	4.5	*Pcdh10*	NM_001098171.1	5300	5331	N13-N20:T_T	-	-0.6	Medium	5.5
20	3'UTR	*POU3F3*	NM_006236.3	4105	4136	N10-N23:C_T	-	-4.3	Medium	4.5	*Pou3f3*	NM_008900.3	4088	4119	N10-N23:C_T	-	-4.3	Medium	4.5
20	3'UTR	*YWHAG*	NM_012479.4	3620	3651	N13-N20:T_T	-	-3.5	High-Medium	6	*Ywhag*	NM_018871.3	3475	3506	N13-N20:T_T	-	-3.5	High-Medium	6
20	3'UTR	*ZBTB20*	NM_001348802.3	7622	7654	-	N23b:C	-6.2	Medium	5.5	*Zbtb20*	NM_001393397.1	8953	8984	N13-N20:T_C	-	-4.4	High	6.5
21	5'UTR	*GNAS*	NM_001410912.1	303	334	-	-	-9	Medium	5	*Gnas*	NM_022000.3	203	234	-	-	-9.7	Medium	5
21	CDS	*AR*	NM_000044.6	3803	3834	-	-	-5	Medium	4.5	*Ar*	NM_013476.4	3639	3670	-	-	-3	Medium	4.5
21	CDS	*ATP6V0D1*	NM_004691.5	747	778	N09-N24:C_C	-	-5.2	Medium	5	*Atp6v0d1*	NM_013477.4	828	859	N09-N24:C_C	-	-7.9	Medium	5
21	CDS	*ATP7A*	NM_001282224.2	4297	4328	N11-N22:T_C	-	-1.3	Medium	4.5	*Atp7a*	NM_009726.5	1762	1793	-	-	-1.1	Medium	5
21	CDS	*CROCC*	NM_014675.5	5143	5174	N09-N24:C_T	-	-7.3	Medium	5	*Crocc*	NM_001145958.2	4871	4902	N09-N24:C_T	-	-4.2	Medium	4.5
21	CDS	*DLG5*	NM_004747.4	262	294	-	N23b:C	-5.1	Medium	5.5	*Dlg5*	NM_001163513.1	497	529	-	N23b:C	-5.1	Medium	5.5
21	CDS	*GSTCD*	NM_001031720.3	1912	1943	N10-N23:G_G	-	-4.4	Medium	4.5	*Gstcd*	NM_001356309.1	2027	2058	N10-N23:G_G	-	-4.4	Medium	4.5
21	CDS	*KCNQ3*	NM_001204824.2	1116	1147	N11-N22:T_T	-	-1.5	Medium	5	*Kcnq3*	NM_152923.3	1848	1879	N11-N22:T_T	-	-1.8	Medium	5
21	CDS	*MAG*	NM_001199216.2	1295	1326	N09-N24:A_C	-	-4.5	Medium	4.5	*Mag*	NM_001346087.2	1891	1923	-	N21b:T	-3.1	Medium	4.5
21	CDS	*MTSS1*	NM_001363298.2	1650	1681	N13-N20:A_A	-	-9.9	High-Medium	6	*Mtss1*	NM_001146180.2	1670	1701	N13-N20:A_A	-	-10.4	High-Medium	6
21	CDS	*MYO5C*	NM_018728.4	2375	2407	-	N23b:C	-1.3	High	6.5	*Myo5c*	NM_001081322.1	2342	2373	N09-N24:T_C	-	-1.3	High-Medium	6

21	CDS	*PLEKHA1*	NM_001377238.1	1113	1144	-	-	-2.1	Medium	5	*Plekha1*	NM_001382378.1	1058	1089	-	-	-2.1	Medium	5
21	CDS	*SEMA5A*	NM_003966.3	2232	2263	N13-N20:A_A	-	-9	Medium	5.5	*Sema5a*	NM_009154.2	2224	2255	-	-	-7.7	High	7
21	CDS	*SMO*	NM_005631.5	2482	2513	N09-N24:T_C	-	-7.2	High-Medium	6	*Smo*	NM_176996.5	2505	2536	N09-N24:T_C	-	-7.2	High-Medium	6
21	3'UTR	*ANKRD40*	NM_052855.4	3948	3979	N10-N23:C_A	-	0	Medium	5	*Ankrd40*	NM_027799.2	3273	3304	N10-N23:C_A	-	0	Medium	5
21	3'UTR	*CPLX2*	NM_001008220.2	3149	3180	-	-	-9	Medium	4.5	*Cplx2*	NM_009946.3	2864	2895	N10-N23:A_C	-	-2.4	Medium	4.5
21	3'UTR	*GRM3*	NM_000840.3	3964	3995	-	-	-7.1	Medium	5	*Grm3*	NM_001417964.1	3876	3907	-	-	-7.1	Medium	5
21	3'UTR	*HEATR1*	NM_018072.6	7541	7572	N11-N22:A_G	-	-0.9	High-Medium	6	*Heatr1*	NM_144835.4	6726	6757	N10-N23:T_T	-	-2.4	Medium	5
21	3'UTR	*RNF214*	NM_001278249.2	1765	1798	N09-N24:T_C	-	0	Medium	4.5	*Rnf214*	NM_001361114.1	1643	1676	N09-N24:T_C	-	0	Medium	4.5
22	CDS	*MOCS2*	NM_004531.5	1049	1082	N10-N23:G_G	-	-5.9	Medium	5.5	*Mocs2*	NM_001113374.2	486	519	N10-N23:G_G	-	-3.5	Medium	5.5
22	CDS	*MORC3*	NM_001320446.2	396	429	-	-	-3.2	Medium	4.5	*Morc3*	NM_001045529.3	431	464	-	-	-3.2	Medium	4.5
22	CDS	*WNT4*	NM_030761.5	1180	1213	N13-N20:G_G	-	-7.6	High-Medium	6	*Wnt4*	NM_009523.2	1050	1083	N13-N20:G_G	-	-7.6	High-Medium	6

<u>Start and End</u>: indicate the range where the IRE is found within the transcript.

<u>Mismatch</u>: indicates the position and type of mismatch present in the IRE (if predicted, otherwise a "-" appears)

<u>Bulge</u>: indicates the position (n20 to n23) and nucleotide of the bulge

<u>Canonical energy</u>: free energy (kcal/mol) of the predicted IRE following IRE constraints as folded by RNAfold (Vienna 2.6.4)

<u>Quality</u>: quality category of the prediction, which can go from Very low, Low, Medium-Low, Medium, High-Medium to High.

<u>Score</u>: score from <0 to 8, where the lower values correspond to bad quality predictions while values close to 8 indicate good quality predictions.

Gold-standard IREs are highlighted in green.

*TFRC/Tfrc contains five IREs within its sequence (A, B, C, D, and E). Of these, IRE B is motif 2, while IREs A, C, D, and E are motif 1. However, for this analysis, we selected only one IRE per gene for the sake of simplicity.

Supplementary Table 5. Detection of literature-reported IREs by SIREs 3.0, RNAAnalyzer and RNAMotif.

Reported IRE in mRNA	SIREs 3.0 (Yes/No, motif)	RNAMotif (Yes/No)	RNAAnalyzer (Yes/No)
FTH [1]	Yes, m1	Yes	Yes
FTL [2]	Yes, m1	Yes	No
TFRC [3]	Yes, m1*4, m2	Yes (5/5 motifs)	Yes, (4/5 motifs)
ALAS2 [4]	Yes, m1	Yes	Yes
dSdhB [5]	Yes, m1	Yes	Yes
ACO2 [6]	Yes, m1	Yes	Yes
Hao1 (Gox) [7]	Yes, m1	No	No
SLC40A1 (FPN1) [8]	Yes, m1	Yes	Yes
NDUFS1 [9]	No	No	No
SLC11A2 (DMT1) [10]	Yes, m1	No	Yes
APP [11]	No	No	No
CDC14A [12]	Yes, m1	No	No
CDC42BPA (MRCKa) [13]	Yes, m2	Yes	No
EPAS1 (HIF2a) [14]	Yes, m1	No	No
SNCA (a-synuclein) [15]	No	No	No
AHSP [16]	No	No	No
BDH2 [17]	Yes, m8	No	No
PFN2 [18]	Yes, m19	No	No
BCL2L1 [19]	Yes, m17	No	No
CD63 [20]	Yes, m2	No	No
Aim2 [21]	No	No	No
CDC42 [22]	Yes, m19	No	No

**% IREs detected**	80.77	46.15	38.46
	(21/26)	(12/26)	(10/26)

References:

1. Hentze, M.W., et al. (1987) A cis-acting element is necessary and sufficient for translational regulation of human ferritin expression in response to iron. Proc Natl Acad Sci U S A, 84, 6730–6734.
2. Aziz, N., and Munro, H.N. (1987) Iron regulates ferritin mRNA translation through a segment of its 5' untranslated region. Proc Natl Acad Sci U S A, 84, 8478–8482.
3. Casey, J.L., et al. (1988) Two genetic loci participate in the regulation by iron of the gene for the human transferrin receptor. Proc Natl Acad Sci U S A, 85, 1787–1791.
4. Dandekar, T., et al. (1991) Identification of a novel iron-responsive element in murine and human erythroid delta-aminolevulinic acid synthase mRNA. EMBO J, 10, 1903–1909.
5. Kohler, S.A., et al. (1995) Succinate dehydrogenase b mRNA of Drosophila melanogaster has a functional iron-responsive element in its 5'-untranslated region. J Biol Chem, 270, 30781–30786.
6. Kim, H.Y., et al. (1996) Identification of a conserved and functional iron-responsive element in the 5'-untranslated region of mammalian mitochondrial aconitase. J Biol Chem, 271, 24226–24230.
7. Kohler, S.A., et al. (1999) Molecular cloning of mouse glycolate oxidase. High evolutionary conservation and presence of an iron-responsive element-like sequence in the mRNA. J Biol Chem, 274, 2401–2407.
8. Abboud, S., and Haile, D.J. (2000) A novel mammalian iron-regulated protein involved in intracellular iron metabolism. J Biol Chem, 275, 19906–19912.
9. Lin, E., et al. (2001) Regulation of the 75-kDa subunit of mitochondrial complex I by iron. J Biol Chem, 276, 27685–27692.
10. Gunshin, H., et al. (2001) Iron-dependent regulation of the divalent metal ion transporter. FEBS Lett, 509, 309–316.
11. Rogers, J.T., et al. (2002) An iron-responsive element type II in the 5'-untranslated region of the Alzheimer's amyloid precursor protein transcript. J Biol Chem, 277, 45518–45528.
12. Sanchez, M., et al. (2006) Iron regulation and the cell cycle: identification of an iron-responsive element in the 3'-untranslated region of human cell division cycle 14A mRNA by a refined microarray-based screening strategy. J Biol Chem, 281, 22865–22874.
13. Cmejla, R., et al. (2006) A novel iron responsive element in the 3'UTR of human MRCKalpha. Biochem Biophys Res Commun, 341, 158–166.
14. Sanchez, M., et al. (2007) Iron-regulatory proteins limit hypoxia-inducible factor-2alpha expression in iron deficiency. Nat Struct Mol Biol, 14, 420–426.
15. Friedlich, A.L., et al. (2007) The 5'-untranslated region of Parkinson's disease alpha-synuclein messenger RNA contains a predicted iron responsive element. Mol Psychiatry, 12, 222–223.
16. dos Santos, C.O., et al. (2008) An iron responsive element-like stem-loop regulates alpha-hemoglobin-stabilizing protein mRNA. J Biol Chem, 283, 26956–26964.
17. Liu, Z., et al. (2012) Siderophore-mediated iron trafficking in humans is regulated by iron. J Mol Med (Berl), 90, 1209–1221.
18. Luscieti, S., et al. (2017) The actin-binding protein profilin 2 is a novel regulator of iron homeostasis. Blood, 130, 1934–1945.
19. Wu, H., et al. (2020) Defective mitochondrial ISCs biogenesis switches on IRP1 to fine tune selective mitophagy. Redox Biol, 36, 101661.
20. Yanatori, I., et al. (2021) CD63 is regulated by iron via the IRE-IRP system and is important for ferritin secretion by extracellular vesicles. Blood, 138, 1490–1503.
21. Robinson, E.K., et al. (2021) Inflammation drives alternative first exon usage to regulate immune genes including a novel iron-regulated isoform. Elife, 10.

22. Shenoy, G., et al. (2023) Iron inhibits glioblastoma cell migration and polarization. FASEB J, 37, e23307.
